# Electrospun Biomaterials for Scarless Acne Wound Healing: Advances and Prospects

**DOI:** 10.3390/jfb16090316

**Published:** 2025-08-29

**Authors:** Jiahui Chen, Liping Zhou, Zhongci Hang, Xiaochun Bian, Tong Huo, Bing Peng, Haohao Li, Yongqiang Wen, Hongwu Du

**Affiliations:** 1 Beijing Key Laboratory for Bioengineering and Sensing Technology, Daxing Research Institute, School of Chemistry and Biological Engineering, University of Science and Technology Beijing, Beijing 100083, China; m202310898@xs.ustb.edu.cn (J.C.); liping-zhou@ustb.edu.cn (L.Z.); zhongci-hang@xs.ustb.edu.cn (Z.H.); xcbian0201@163.com (X.B.); huotong1987@163.com (T.H.); pbing0217@163.com (B.P.); m202220943@xs.ustb.edu.cn (H.L.); 2 Shandong Laboratory of Advanced Materials and Green Manufacturing at Yantai, Yantai Zhongke Research Institute of Advanced Materials and Green Chemical Engineering, Yantai 264006, China

**Keywords:** acne, acne scars, electrospinning, nanofibers, stem cells, exosomes

## Abstract

Acne vulgaris is a chronic disease that occurs in the pilosebaceous units and ranks eighth in the global prevalence of all diseases. In its severe forms such as pustules, cysts, and nodules, acne can lead to permanent scarring and post-inflammatory hyperpigmentation, which are often difficult to reverse in the short term and significantly affect patients’ psychological well-being and social interactions. Although a variety of pharmacological treatments are available, including retinoids, antibiotics, anti-androgens, benzoyl peroxide, and corticosteroids, the high recurrence rate and limited efficacy in scar prevention highlight the urgent need for innovative therapeutic strategies. Electrospinning technology has recently gained attention for fabricating nanofibrous patches with high porosity, biocompatibility, and biodegradability. These patches can offer antibacterial activity, absorb exudates, and provide mechanical protection, making them promising platforms for acne wound care. This review first outlines the pathophysiology of acne and the biological mechanisms underlying scar formation. We then present an overview of electrospinning techniques, commonly used polymers, and recent advancements in the field. Finally, we explore the potential of electrospun nanofibers loaded with mesenchymal stem cells or exosomes as next-generation therapeutic systems aimed at promoting scarless acne healing.

## 1. Introduction

Acne vulgaris is a common inflammatory disease of the hair follicle and sebaceous gland units, primarily affecting areas with the highest density of these units, such as the face, neck, upper chest, shoulders, and back. Statistics show that approximately 45 million people worldwide are affected by acne to varying degrees [1], with the prevalence of acne in adolescents estimated to be between 28.9% and 91.3% [2]. The age range of acne is relatively wide, mainly concentrated in adolescence (10–19 years old), early adulthood (20–30 years old), and middle-aged and elderly people (over 40 years old), with the highest incidence rate in adolescence, at about 80–90% [3]. Due to its long course and tendency for recurrent outbreaks [4], and the formation of disfiguring hypertrophic or atrophic scars during the healing process, acne ranks third among chronic skin diseases that cause disability [5]. Additionally, the changes in appearance caused by acne lead to negative psychological and social impacts, significantly affecting the quality of life. Compared to non-acne sufferers, those with acne have higher rates of anxiety, low self-esteem, and depression [6]. Therefore, finding an effective acne treatment with minimal side effects is urgently needed, not only to alleviate pain and repair the facial appearance of patients but also to reduce their psychological burden and restore their self-worth and sense of identity.

**Figure 1:**
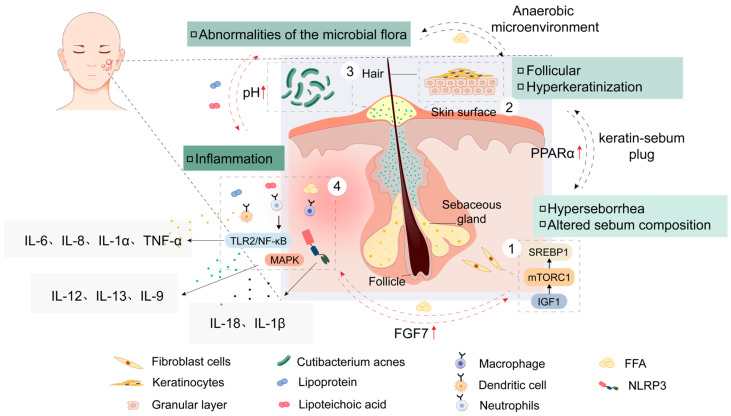
Summary of the Main Causes of Acne Occurrence. Including 1. Excessive sebum secretion and compositional changes caused by upregulation of androgens, 2. Excessive keratinization of hair follicles, 3. Abnormal microbial community, and 4. Inflammation. There is a mutually constraining or inducing relationship between these four core factors, indicated by dashed arrows in the figure. The red solid arrow represents the upward adjustment of a certain factor or indicator.

Before proposing new acne treatment strategies, it is necessary to understand the close relationship between acne occurrence and scar formation. Simply put, the duration and severity of inflammation can alter the structure of the sebaceous glands to some extent, leading to the formation of atrophic scars. The exact mechanisms of acne development are not yet fully understood, but the medical community generally agrees on four main factors: increased sebum secretion, hyperkeratinization of hair follicles, disruption of the skin surface microbiome, and subsequent inflammatory responses [7]. Regarding the formation of acne scars, current research mainly focuses on transforming growth factor-β (TGF-β) secreted by fibroblasts and other cells, which leads to scarring through inducing myofibroblast differentiation, extracellular matrix (ECM) synthesis, fibroblast proliferation, and migration [8] (Figure 1). Therefore, to address the physical and psychological trauma caused by scars in acne patients, the development of new strategies should focus on inhibiting and intervening the excessive inflammation caused by acne.

Today, electrospinning technology is one of the most traditional methods for preparing continuous fibers. Its scalability, repeatability, ease of use, and multifunctionality make it the preferred nanofiber production method, showing strong development potential in fields such as water purification, food packaging, tissue engineering [9], biomedicine [10], healthcare, and cosmetics [11]. The primary application form of nanofibers in skin treatment is patches, which have shown excellent therapeutic effects on burn wounds, diabetic wounds, and ulcerative lesions [12]. Recent studies have attempted to leverage nanofibers’ porous structure and good biocompatibility for acne treatment [13,14,15]. An ideal nanofiber patch can facilitate good gas exchange between the acne-affected area and the external environment, and act as a carrier for therapeutic drugs, providing long-term treatment for acne through localized slow release. Mesenchymal stem cells are the most extensively studied cell type in stem cell therapy, capable of participating in tissue repair and local functional recovery. Their immunomodulatory effects have also made them a research hotspot in regenerative medicine [16]. Currently, most approaches using electrospinning technology to address acne focus primarily on antibacterial effects and drug release [14,17]. In contrast, we aim to explore a nanofiber strategy that utilizes mesenchymal stem cells (MSCs) or exosomes to achieve scarless acne repair by inhibiting inflammation and downregulating scar formation-related factors.

To date, the potential of electrospun fibers as carriers for MSCs or exosomes to modulate inflammation and minimize scar formation remains underexplored. This review aims to (i) elucidate the biological basis of acne and scar formation; (ii) provide an overview of electrospinning technologies and the advantages and potential of electrospun fibers in improving acne scars compared to previous treatments; and (iii) critically assess the application of stem cell- or exosome-loaded nanofibers in promoting scarless healing. By highlighting current challenges and future opportunities, we hope to offer new perspectives for the development of multifunctional electrospun systems in acne therapy.

In this review, we first briefly discuss the causes of acne and provide a detailed and comprehensive overview of the widely accepted mechanisms underlying acne development in current research. We then examine the molecular mechanisms involved in the formation of atrophic and hypertrophic scars during the acne healing process, along with the principles and current clinical treatment options. Unlike these treatment methods, our goal is to propose a proactive intervention strategy aimed at achieving scar-free acne repair before scar formation occurs. To this end, we focus on electrospinning technology, highlighting its advantages and potential in improving acne scarring compared to conventional treatments. In the final section of the review, we propose incorporating mesenchymal stem cells or exosomes with anti-inflammatory and anti-scarring properties into electrospun nanofibers. We critically summarize the feasibility and limitations of this approach, discuss the challenges facing the clinical translation of exosome-loaded electrospun nanofibers, and propose various strategies for the personalized customization of future nanofiber patches. This provides new insights and directions for further exploration of electrospinning technology in acne therapy.

## 2. Formation of Acne Scars

### 2.1. Mechanisms of Acne Occurrence

In addition to genetic factors, mental stress and emotional disturbances causing endocrine disorders, diet and lifestyle (consumption of high-sugar, high-fat, and spicy foods, staying up late), and improper use of cosmetics [18,19], the mechanisms of acne development can be broadly categorized into four types: androgen-mediated increased sebum secretion, hyperkeratinization of hair follicles [20], imbalance of the skin microbiome, and subsequent activation of inflammatory cascade reactions [21]. In the past, researchers generally believed that the overgrowth of *Propionibacterium acnes* was the cause of inflammation and infection [22], but later studies gradually revised this conclusion. The *Propionibacterium acnes* on the skin surface exists in various subspecies, and when this diversity is lost, it triggers the activation of the innate immune system, leading to skin inflammation [23,24,25].

#### 2.1.1. Pathways Involving Increased Sebum Secretion Mediated by Androgens

During puberty, the secretion of growth hormone, insulin-like growth factor 1 (IGF-1), and androgens increase physiologically, which may be caused by various factors [26], such as obesity, genetic factors, environmental stress, and chronic sleep deprivation. This period also coincides with the peak of sebum secretion and acne occurrence. Studies have shown that acne is an androgen-dependent sebaceous gland disease driven by insulin-like growth factor 1 (IGF-1) [27]. The synthesis of androgens is crucial for excessive sebum secretion and sebaceous gland proliferation [28], and an increase in sebum secretion may directly lead to the onset of acne [21]. In a clinical trial, subjects were treated with either IGF-1 deficiency or IGF-1 administration. The results indicated that IGF-1 deficiency helps inhibit the occurrence of acne [29]. IGF-1 stimulates increased adrenal sensitivity to adrenocorticotropic hormone (ACTH), inducing testosterone synthesis, which is then reduced to dihydrotestosterone (DHT) by 5α-reductase expressed in sebocytes [30]. Androgen receptors are expressed in the nuclei of early differentiating sebocytes [31] and in dermal fibroblasts. DHT regulates keratinocyte differentiation by modulating dermal fibroblasts. An experiment investigating the relationship between androgens and keratinocytes demonstrated that androgens alter keratinocyte differentiation by enhancing dermal fibroblast growth factors, including amphiregulin, epiregulin, fibroblast growth factor 10, and insulin-like growth factor-binding proteins [32].

**Figure 2:**
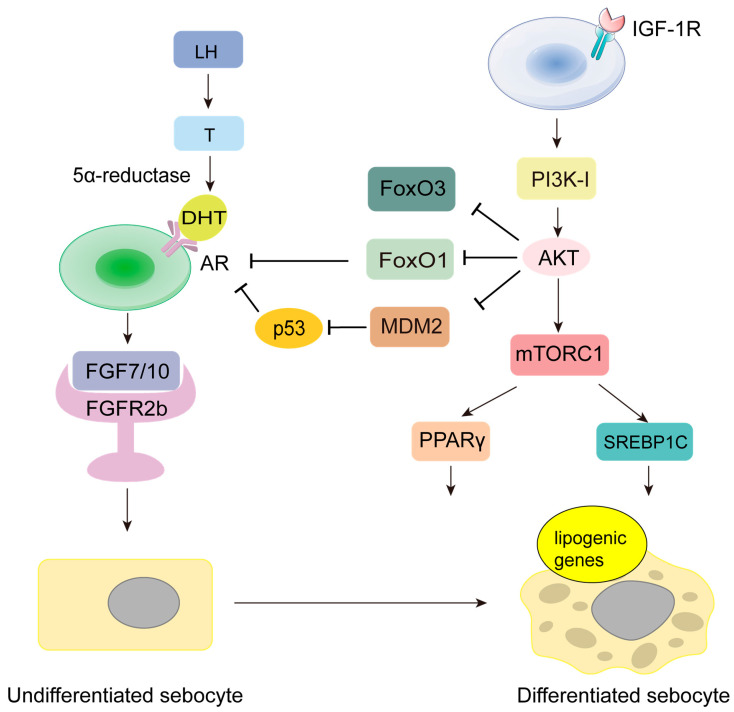
Signaling pathways involving androgens and insulin-like growth factors in the differentiation of sebocytes and lipid accumulation.

Another study used fibroblast growth factor receptor (FGFR) antagonists to inhibit its signaling, ultimately finding that sebocyte proliferation and sebum accumulation were reduced accordingly [33], corresponding to the previous experimental results. The IGF-1/PI3K/AKT signaling pathway promotes the development of acne, with AKT activation prompting the phosphorylation of the FoxO1 nuclear transcription factor, reducing its nuclear presence and relieving its inhibitory effect on androgen receptors [34]. Additionally, the literature reports that AKT can promote acne development through the downstream MDM2/p53 pathway [35] and enhance the activity of the mTORC1 target, thereby promoting the expression of sterol regulatory element-binding protein-1c (SREBP-1c) and peroxisome proliferator-activated receptor gamma (PPAR-γ) [36]. Androgen receptor signaling activates PPARγ, upregulating the expression of sebum-producing genes, while Wnt signaling and FoxO nuclear transcription factors inhibit it. In summary, androgens influence keratinocyte differentiation through their effects on fibroblasts, altering sebocyte differentiation and sebum synthesis (Figure 2).

#### 2.1.2. Excessive Keratinization of the Follicular Sebaceous Duct

Excessive follicular keratinization is a major factor in the pathogenesis of acne, and it is also the primary target for vitamin A-like treatments for acne. In normal hair follicles, keratinocytes shed and are transported to the skin surface with the flow of sebum. However, when follicular cells become sticky and keratinocyte proliferation accelerates, the balance between shed cells and those transported to the skin’s surface is disrupted, leading to the formation of microcomedones [37]. Researchers have found that the keratinization pattern in the follicles of acne patients changes with the spontaneous variations in keratinocytes, such as increased keratin density, faster keratinocyte division, and increased tension fibers and bridge granules in the follicular funnel region, resulting in aggregation [38]. There are many causes of excessive follicular keratinization, including changes in sebum composition, bacterial metabolites, inflammatory mediators, and androgen levels. For instance, a decrease in linoleic acid levels can lead to the disruption of the epithelial barrier function or a lack of epidermal lipids in the follicles [39]. Steroid sulfatase is an enzyme that converts cholesterol sulfate into cholesterol, and a reduction in this enzyme can also lead to excessive follicular keratinization. Additionally, androgens and pro-inflammatory factors such as IL-1β and its receptor, as well as receptor antagonists [40], are also considered to be related to excessive follicular keratinization in some studies [41].

#### 2.1.3. Disruption of Microbial Community Homeostasis

The human skin surface microbiome is a complex community of bacteria, fungi, and viruses. In healthy skin, the microbiome is in a state of equilibrium, with different microbial species forming symbiotic relationships with the skin and working together with the immune system to create a robust biological barrier that protects the skin from external threats [42]. The skin surface forms various microenvironments depending on factors such as pH, temperature, humidity, sebum content, and topography [43]. *Corynebacterium*, *Staphylococcus*, and *Bacillus* are among the most common bacterial genera on the skin and exhibit distinct skin environment-specific characteristics. For example, *Propionibacterium* is a lipophilic genus, whereas *Staphylococcus* and *Bacillus* are more abundant in the skin’s moist areas [44]. *Cutibacterium acnes* (*C. acnes*), *Corynebacterium*, *Bacillus*, *Staphylococcus*, *Pseudomonas*, and *Malassezia* co-exist in the sebaceous glands of hair follicles [43].

The traditional belief that *C. acnes* infection is a major pathogenic factor in acne development is widely known and accepted, even though *C. acnes* is also commonly found in healthy skin [45]. In fact, as research into acne has advanced, new findings related to the microbiome have somewhat deviated from previous theories. There has not been direct and compelling evidence to show that the proliferation of *C. acnes* is proportional to the severity of acne. Metagenomic analyses indicate that the abundance of *C. acnes* does not differ significantly between acne patients and healthy skin [46,47]. Moreover, *C. acnes* can induce the synthesis of lipids such as triglycerides, ceramides, cholesterol, and free fatty acids, which play an essential role in epithelial barrier formation and microbial balance [48]. The theory of *C. acnes* overgrowth leading to acne being overturned has been replaced by the concept of imbalanced *C. acnes* developmental system types. It has been established that *C. acnes* types I and II are more common in healthy skin, while developmental system type IA1 is associated with acne [25]. A study using NGS technology to analyze the skin microbiome of acne patients found that the dominant microorganisms were *Corynebacterium*, *Staphylococcus*, and *Malassezia*. It also discovered that the metabolic activity of the microbiome in acne comedones was significantly higher than that on the skin surface, producing many enzymes involved in inflammation and comedo formation: lipases, phosphatases, neuraminidases, proteases, and hyaluronidases [49]. Other research also shows that *Staphylococcus* is more abundant than *Corynebacterium* on the surface of comedones, papules, and pustules in some acne patients [50]. With the emergence of the concept that follicular sebaceous units can produce biofilms, there is a deeper understanding of the microbiome on the skin surface of acne patients [51,52]. Biofilms provide a protective barrier for a large number of bacteria, helping them survive in adverse environments. The development of acne is related to changes in the skin surface microbiome, which replaces the previously stable skin microbiome of childhood, with increased numbers of *C. acnes* and *Bacillus* in the new skin microbiome [53].

#### 2.1.4. Inflammatory Response

**Figure 3:**
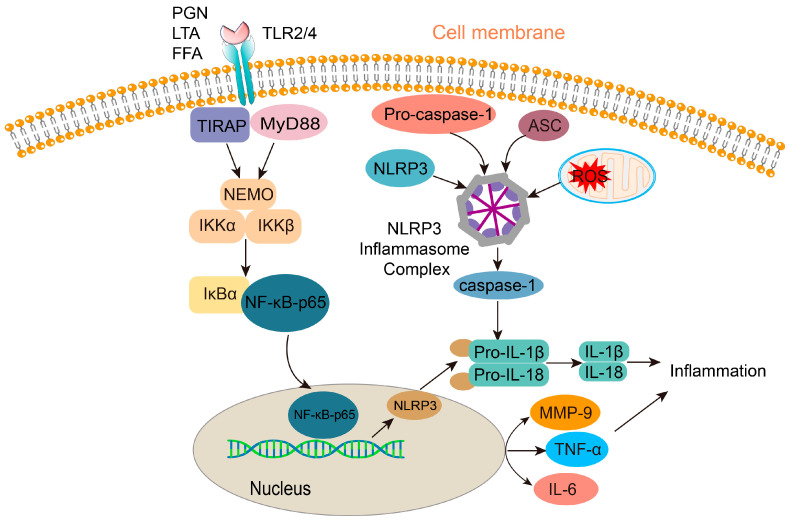
Acne inflammation signaling pathways mediated by lipoteichoic acid, peptidoglycan, and short-chain fatty acids lead to various inflammatory factors production.

*C. acnes* has been shown to promote and enhance the inflammatory response by interacting with Toll-like receptor 2 (TLR-2) or Toll-like receptor 4 (TLR-4) on keratinocytes through its potential ligands [54], leading to the induction of pro-inflammatory factors such as TNF-α, IL-1β, IL-6, IL-8, IL-12, and various chemokines [55]. One study demonstrated that when keratinocytes were treated with peptidoglycan (PGN) and lipoteichoic acid (LTA) from *C. acnes*, TLR-2 on the keratinocytes could be activated, subsequently stimulating neutrophils to secrete TNF-α and IL-8. These chemokines’ release triggers the NF-κB signaling pathway [56]. Another study simulated the microenvironment of hair follicles during acne development by culturing *C. acnes* in an occluded environment characterized by lipid richness and low oxygen. In this environment, *C. acnes* produces short-chain fatty acids (SCFAs) and enhances the expression of pro-inflammatory factors through epigenetic mechanisms [57]. The NOD-like receptor pyrin domain-containing 3 (NLRP3) signaling pathway has also been implicated in the inflammatory response associated with acne. The NLRP3 inflammasome, typically composed of NLRP3, the adaptor protein ASC, and the effector protein pro-caspase-1 [58,59], can be triggered by *C. acnes*, leading to the release of mature caspase-1. Caspase-1 then converts pro-IL-1β to mature IL-1β and its subsequent release [60,61]. Recent research has also highlighted the important role of Th17 cell-mediated adaptive immune responses in acne development [62]. *C. acnes* triggers TLR-2, which through signaling pathways stimulates sebocytes to produce transforming growth factor-β (TGF-β), inducing the differentiation of CD4+ naïve T cells into Th17 cells, which then release a range of pro-inflammatory factors including IL-17, IL-21, IL-22, and IL-23 [63,64] (Figure 3).

### 2.2. Acne Scars

#### 2.2.1. Mechanisms of Acne Scar Formation

Acne can lead to scarring if not perfectly healed, with scars primarily classified into atrophic and hypertrophic types. The process of scar formation is closely linked to the preceding inflammatory response. Interestingly, not every person with acne will ultimately develop scars. The specific mechanisms underlying hypertrophic scar formation are not yet fully understood, but excessive fibroblast proliferation, the deposition of extracellular matrix, and collagen are well-known major contributors [65]. Research indicates that the formation of hypertrophic scars is associated with several pathways, including TGF-β1/Smad, PI3K/AKT, and JAK/STAT3 [66,67]. TGF-β plays a crucial role in the formation of hypertrophic scars, as TGF-β1 stimulates fibroblast proliferation and collagen formation while inhibiting extracellular matrix degradation [66]. Conversely, TGF-β3 can counteract excessive collagen production [68]. Studies have shown that increased inflammation raises the concentration of fibrogenic cytokines such as TGF-β, platelet-derived growth factor (PDGF), and interleukin-4 (IL-4), leading to the development of hypertrophic scars [69].

Atrophic scars are more common than hypertrophic scars among acne patients [70], and researchers have focused more on studying and treating atrophic scars. The mechanisms involved are also related to the TGF-β/Smad pathway. An experiment centered on TGF-β1 investigated its role in atrophic scar formation. The study found that in the downstream pathways of TGF-β1, NF-κB signaling is activated through TGF-β-activated kinase 1 (TAK1), which then transcribes inflammatory factors such as TNF-α and IL-1β, and promotes the synthesis of various matrix metalloproteinases (MMPs), exacerbating the degradation of collagen, elastin, and extracellular matrix. Additionally, the investigation revealed a significant decrease in epidermal proliferation, which was hypothesized to be related to the downregulation of C-Myc (a transcription factor promoting cell cycle progression) and phosphorylated extracellular signal-regulated kinases. These findings suggest that early intervention and suppression of the inflammatory response in acne can effectively reduce or even eliminate the likelihood of developing hypertrophic or atrophic scars [71].

#### 2.2.2. Clinical Treatment Methods for Acne Scars

Currently, common clinical treatments for acne scars include pulsed dye laser (PDL) [72], platelet-rich plasma (PRP) injection [73], fractional carbon dioxide (CO_2_) laser [74], photodynamic therapy (PDT) [75], corticosteroid injection, topical application of azelaic acid or oral administration of tretinoin [76]. The primary mechanism of pulsed dye laser therapy is selective photothermolysis, which penetrates the dermis and destroys dilated blood vessels in the affected areas, thereby cutting off the nutrient supply to pathological tissues [77]. This method is known for its high safety and minimal side effects. Fractional CO_2_ laser promotes skin repair and collagen remodeling by using localized heating to vaporize skin tissue and stimulate collagen proliferation [78]. However, due to uneven energy distribution and poor laser penetration, this method may lead to adverse reactions such as tissue damage or pigmentation [79]. The principle of photodynamic therapy involves applying a photosensitizer to the lesion area, which, after absorbing a large amount of light energy, becomes activated and generates large quantities of singlet oxygen and reactive oxygen species. This induces oxidative stress-mediated apoptosis or necrosis of local cells or tissues, thereby inhibiting the excessive proliferation and accumulation of fibroblasts. We reviewed eight clinical studies conducted over the past three years on the use of various types of fractional lasers for treating acne scars [80,81,82,83,84,85,86,87] (Table 1). Some studies compared the effects of single-session CO_2_ fractional laser with combination therapies such as ALA-PDT, PRP, and isotretinoin, providing supportive data and conclusions regarding the efficacy and safety of laser-based acne scar treatments. Although a variety of treatment methods for acne scars have been developed and established as main strategies for scar management, their overall efficacy is still not absolute and may be accompanied by adverse effects and side effects, leading to secondary injury in patients. Other commonly used clinical treatments also have inherent limitations. For example, corticosteroid injections are only applicable to hypertrophic scars, and repeated high-dose injections can damage and depress dermal collagen and adipose tissue. The associated pain from injections also reduces patient compliance. Azelaic acid and retinoic acid can cause significant irritation in the early stages of use. Moreover, azelaic acid has limited efficacy in scar treatment, while retinoic acid is more effective for improving atrophic scars. Overall, there is a clear need to identify strategies that offer significant therapeutic benefits with minimal side effects.

## 3. Electrospinning Technology in Acne Treatment

### 3.1. Electrospinning Technology

#### 3.1.1. Principles of the Technology

**Figure 4:**
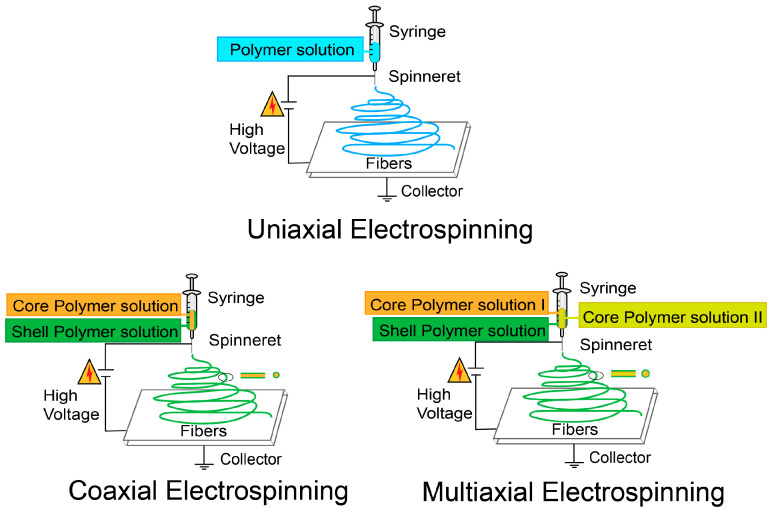
Schematic diagrams illustrating different types of electrospinning technologies.

The primary principle of electrospinning is to use electrostatic forces to convert a polymer solution into continuous polymer fibers with diameters ranging from micrometers to nanometers (40–2000 nm) [88,89]. The main process involves placing a liquid or melt between two conductors with opposite polarities, and applying a high-voltage field to charge the liquid or melt. Under the combined effects of electrostatic force, viscosity, and surface tension, the solution forms a jet that ejects from the tip of the needle and extends toward the counter electrode. As the solvent evaporates, nanofibers are deposited onto a collector, forming a porous structure with similar mechanical properties in both the longitudinal and transverse directions [90]. The key components of an electrospinning device include the pump for driving the polymer solution through the syringe, the high-voltage power supply, and the collector for retrieving the nanofibers [91]. The final morphology of electrospun fibers is influenced by the properties of the solution (concentration, viscosity, molecular weight, conductivity, surface tension), process parameters (voltage, flow rate, collector type, distance between the needle tip and collector), and environmental parameters (temperature, humidity) [9,92]. Advanced electrospinning technologies are classified into two types based on the spinneret configuration: needle-based and needleless. Needle-based electrospinning technologies include single-needle electrospinning, coaxial electrospinning, tri-axial and multi-axial electrospinning, centrifugal electrospinning [93], and 3D electrospinning [94]. Needleless electrospinning technologies include roller, bubble, corona, wire, and high-speed electrospinning [95] (Figure 4).

#### 3.1.2. Common Materials and Characteristics of Nanofibers

The materials used to prepare nanofibers can be classified into three main categories based on their sources and compositions: natural polymers, synthetic polymers, and polymer mixture of natural and synthetic polymers [96].

Some natural polymers are favored by researchers in the field of electrospinning due to their various properties such as low cost, non-toxicity, modifiability, biodegradability, bioactivity, and biocompatibility [97]. Including gelatin, hyaluronic acid, collagen, and polysaccharides like cellulose, chitosan, pectin, dextran, starch, and alginates [98]. The most commonly used materials are dextran, sodium alginate, and hyaluronic acid. However, most natural polymers have disadvantages such as insufficient mechanical strength and poor stability. When used as wound dressings, possible measures may need to be taken to regulate their degradability, water absorption, and mechanical support capacity. The viscosity, conductivity, molecular weight, surface tension, and solvent are some factors affecting the electrospinning performance of polymers [99].

Among synthetic polymers, poly(ethylene oxide) (PEO), poly(ε-caprolactone) (PCL) [100,101], poly(lactic acid) (PLA) [102], poly(D, L-lactic-co-glycolic acid) (PLGA) [103,104], polyurethane (PU) [105], and polyacrylonitrile (PAN) [106,107] are also commonly used for electrospinning. These materials can provide the necessary mechanical strength, stability, and durability [108], making them suitable for drug delivery, wound dressings, and vascular and organ implants. However, their hydrophobicity [109] and lower biocompatibility are detrimental to cell adhesion and proliferation [110], which need to be addressed.

Researchers have developed polymer mixture of natural and synthetic polymers to overcome the limitations of both natural and synthetic polymers in the production of nanofibers. In brief, this involves combining natural and synthetic polymers to produce electrospun fibers, thereby modifying their properties to address individual limitations. The resulting hybrid materials can simultaneously offer the excellent biocompatibility of natural polymers and the high mechanical strength and stability of synthetic polymers [111,112]. This approach has been applied in the development of a hybrid scaffold made from poly(ε-caprolactone) (PCL) and hyaluronic acid (HA), which incorporates short self-assembling peptides FmocFRGD and exhibits a similar morphology to the extracellular matrix, effectively promoting osteogenesis [113].

In recent years, numerous studies have emerged on using nanofiber hydrogels as 3D cell culture platforms [114,115,116]. Researchers have successfully improved the uniform and ordered structure of traditional hydrogels by incorporating nanofibers [117], thereby more accurately mimicking the complex architecture of native tissues [118]. In one study, a nanofiber-reinforced hydrogel composite was prepared using coaxial electrospinning to fabricate core–shell nanofibers from natural proteins—zein and gelatin. These hydrated nanofibers were then transformed into reinforced hydrogels. The presence of nanofibers significantly enhanced the physical integrity of the hydrogel and imparted the mechanical strength and toughness of the fibers [119]. Another study found that adjusting the nanofiber content could modulate the stiffness of the extracellular matrix, thereby directing the differentiation of adipose-derived mesenchymal stem cells [120]. In addition, multidimensional nanofibers obtained by integrating fibrin, polycaprolactone (PCL), and alginate have been shown to support the proliferation and angiogenesis of human umbilical vein endothelial cells, showing promising potential in wound healing and tissue regeneration [121].

### 3.2. Electrospun Fibers for Acne Treatment

#### 3.2.1. Potential Advantages

Nanofibers can be classified into several types based on their morphology: non-porous, mesoporous, hollow, and core types. Among these, porous fibers [122], spiral fibers [123], composite fibers [124], branched nanofibers, smooth nanofibers, core–shell nanofibers, and banded nanofibers are some common fiber shapes. In addition to their high porosity and lightweight characteristics, nanofiber membranes exhibit various excellent functions, such as filtration performance, mechanical properties, elasticity, heat resistance, and chemical resistance. From both structural and functional perspectives, electrospun fibers offer promising potential for scarless acne treatment.

##### Structural Advantages

**Figure 5:**
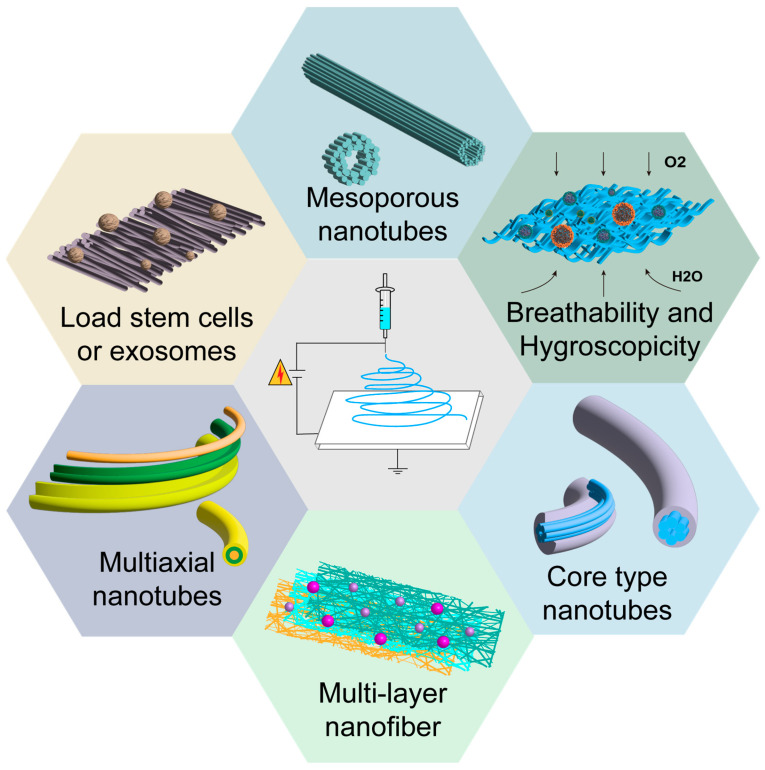
Different morphologies of electrospun nanofibers.

The diversity in the morphology of electrospun fibers is the basis for their excellent stretchability. When used in the development of acne treatment patches, electrospun fibers can provide a closer fit to the skin. Compared to some acne patches based on gels or non-woven fabrics, electrospun nanofibers offer high moisture-wicking properties and superior breathability. When tissue fluid exudates occur on the surface of inflammatory acne lesions, it can be immediately absorbed by the electrospun fibers, keeping the acne area dry and effectively preventing conditions that favor the proliferation of bacteria. Furthermore, good breathability ensures timely oxygen delivery to the inflamed area, preventing hypoxia that worsens the inflammation [125]. The unique high surface area to volume ratio of electrospun nanofibers allows them to have a high drug loading rate, laying a solid structural foundation for the sustained and controllable release of active ingredients. At the same time, the porous morphology of the fibers endows electrospun fibers with the capability to deliver and release drugs in a controlled manner. Currently, topical treatment is a first-line approach for acne, with commonly used medications including retinoids and isotretinoin, clindamycin, tetracycline, benzoyl peroxide, and erythromycin [126,127]. Effective drug delivery and penetration through the stratum corneum are key challenges in treatment, and researchers have been dedicated to finding methods to efficiently deliver drugs to target areas and enhance drug absorption at the skin surface. As research continues, scientists are turning their attention to nanofibers, which are considered to have significant potential for drug delivery [128]. Nanofibers, with diameters ranging from micrometers to nanometers, can be viewed as nanostructures in fiber form, capable of effectively delivering both hydrophilic and hydrophobic substances. Their modifiability provides multifunctionality in biological therapies [129]. Specific release profiles that suitable for different types of drugs can be designed by adjusting the diameter, shape, porosity of the nanofibers, as well as the ratio of drugs to polymers. Some researchers have already developed various patches using nanofibers as carriers and different antibacterial agents as therapeutic drugs, achieving promising results in preliminary experiments [130] (Figure 5).

##### Functional Advantages

When common acne progresses to more severe stages such as pustules and nodules, the excessive inflammation and severe skin damage can lead to the formation of difficult-to-remove scars during the later stage recovery process. Therefore, consciously and effectively intervening and suppressing scar formation during the acne recovery and treatment period can greatly prevent this phenomenon. Hypertrophic scarring involves complex mechanisms and pathological processes of various cells, with the continuous activation of fibroblasts and their excessive differentiation into myofibroblasts being key factors in this phenomenon [131]. To target the suppression of fibroblast differentiation into myofibroblasts, pressure therapy is a cost-effective clinical treatment method. Its main principle is to minimize mechanical tension to inhibit the formation of hypertrophic scars.

After pressure therapy on scars, their structure becomes similar to that of normal skin, showing a thinner epidermis, orderly and loosely distributed collagen, and retained elastic fibers [132]. The specific mechanism of pressure therapy involves the proliferation and migration of proliferative fibroblasts. The application of pressure can effectively block key checkpoints in their cell cycle (G2/M and S phases), promoting fibroblast apoptosis [133]. Additionally, pressure can alter the metabolic pathways of fibroblasts, affecting their proliferation and collagen synthesis ability by enhancing glycolysis and fatty acid synthesis [134]. In addition to its effects on fibroblasts, pressure therapy can also regulate the synthesis of collagen synthesis or degradation enzymes. It achieves this by appropriately downregulating collagen synthase expression while enhancing matrix metalloproteinase activity to accelerate the collagen remodeling process [135]. Evidence suggests that pressure therapy can induce a hypoxic environment and reduce the expression of TGF-β, thereby inhibiting the TGF-β-Smad2/Smad3 signaling pathway and limiting fibroblast activation [136].

In another clinical study, pressure therapy was shown to dedifferentiate myofibroblasts from human scars into normal fibroblasts, thereby reducing scar formation [137]. This process involves the integrin β 1/ILK signaling pathway, which can convert mechanical and chemical signals of the extracellular matrix (ECM) into intracellular signals, playing a key role in controlling cell adhesion, proliferation, survival, migration, and differentiation. The activity of ILK directly or indirectly maintains the function of β-catenin/TCF-4. TCF-4 is a key downstream transcription factor used to maintain the transcription level of SMYD3. H3K4me3 is responsible for accumulating in the promoter region of the ITGBL1 gene, which expresses the phenotype of myofibroblasts, promoting its transcription. SMYD3 is a methyltransferase of H3K4me3 [138]. Specific experiments have shown that after pressure treatment, the integrin β 1/ILK pathway in scar-derived myofibroblasts is inhibited, leading to a decrease in TCF-4 levels. This, in turn, reduces SMYD3 expression and H3K4 trimethylation (H3K4me3) levels, further inhibiting ITGBL1 expression, ultimately leading to the dedifferentiation of myofibroblasts into fibroblasts.

Electrospun nanofiber membranes can achieve high mechanical performance and good elasticity by altering their structural morphology during preparation. These properties can apply appropriate pressure to the acne-affected areas, inducing the new fibroblasts in the damaged skin to grow in an orderly direction rather than excessive or disordered differentiation into myofibroblasts, thus assisting the acne healing process toward scar-free recovery. Although there are currently no clinical trials exploring the mechanisms of electrospun fibers suppressing hypertrophic scarring caused by acne through mechanical pressure, this therapeutic approach is theoretically feasible and significant. It provides valuable insights and new approaches for researchers developing nanofiber patches for acne treatment.

Since the mechanical strength and toughness of nanofibers are critical for their application in pressure therapy, we next discuss several strategies to enhance the overall strength by improving intrinsic properties of the nanofibers, such as fiber alignment, fiber diameter, porosity, and functional groups, thereby expanding their potential in pressure-based treatments. One approach is to select materials that inherently possess strong mechanical properties—such as polycaprolactone (PCL), polylactic acid (PLA), polyurethane (PU), and polyethylene terephthalate (PET)—as the backbone structure of nanofibers. Blending two or more of these polymers can also improve the comprehensive mechanical performance of the fibers [139,140]. Properly increasing the polymer concentration and molecular weight of the spinning solution can help form more continuous fibers, thereby improving their tensile strength and modulus. In addition, optimizing parameters such as applied voltage and collection distance can effectively regulate the stretching and arrangement of fibers. Using inorganic or organic nanomaterials as fillers is another strategy to enhance mechanical properties. The diameter of nanofibers directly affects their overall mechanical properties, and a medium diameter can ensure structural integrity and flexibility [141]. Studies have shown that different diameters of nanofibers have an impact on the migration ability of human skin fibroblasts. Fibers with larger diameters (3000 nm) can significantly increase the migration speed and related gene expression of cells [142]. In addition, another study investigated the polarization of vascular endothelial cells and activation of the Rac1/Cdc42 signaling pathway by constructing nanofibers with different surface micro topological structures. The complex nano three-dimensional surface structure is more conducive to enhancing migration [143].

Increasing the polymer concentration and molecular weight of the spinning solution appropriately helps form more continuous fibers, thereby enhancing their tensile strength and modulus. In addition, optimizing parameters such as applied voltage and collecting distance can effectively regulate fiber stretching and alignment. Incorporating inorganic or organic nanomaterials as fillers is another strategy to reinforce mechanical properties. The diameter of nanofibers directly affects their overall mechanical performance, and a moderate diameter can ensure both structural integrity and flexibility.

#### 3.2.2. Research Progress

The storage and absorption efficiency of drugs in the skin are key evaluation factors for local delivery systems. For drugs that do not achieve good permeability through direct application, new delivery systems need to be developed. The involvement of electrospinning technology has effectively addressed this issue, with nanofibers showing promising results in loading high concentrations of drugs and localized transdermal delivery.

##### Essential Oils or Plant Extracts

Wael Mamdouh and colleagues developed polyvinyl alcohol/quercetin/essential oil composite nanofibers using single-axis electrospinning. This system addressed the disadvantages of quercetin, such as poor water solubility, epidermal permeability, and stability when used directly on the skin, while fully utilizing its antibacterial, anti-inflammatory, and antioxidant properties. The nanofibers produced under optimal parameters were smooth and cylindrical, with no bead formation, indicating that the loaded drug was well and totally mixed into the single fibers. Experimental results showed that the quercetin loading rate in the nanofibers reached 96% ± 0.006 and effectively penetrated the patient’s skin. The reduction in inflammatory lesions was 61.2% ± 10.2%, while the reductions in acne and total skin lesions were 14.7% ± 16.5% and 52.9% ± 9.9%, respectively, proving this to be a promising nanofiber dressing [144]. Lavender and peppermint are two natural plants that can be extracted for their antibacterial essential oils, but their activity decreases significantly during use, which is a major drawback. Electrospinning technology provides a powerful encapsulation platform, enhancing essential oil transdermal absorption while mitigating its degradation rate. After the addition of lavender and peppermint essential oils, the nanofibers exhibited a curved morphology, and their flexibility changed. Subsequent tests showed encouraging results in terms of both cytotoxicity levels and inhibition rates against *C. acnes* and *Staphylococcus epidermidis*, with minimum inhibitory concentrations (MIC) and minimum bactericidal concentrations (MBC) ranging from 5.7 to 9.4 µL/mL and 9.4 to 25.0 µL/mL, respectively [145].

Another study used a similar method to prepare polycaprolactone nanofiber/peppermint oil patches. When adjusted to the optimal concentration, the peppermint oil nanoemulsion was completely encapsulated in the polycaprolactone fiber pores, showing clear, randomly oriented beads and smooth, continuous fiber morphology, with significant antibacterial activity against various fungi and bacteria [146]. Tang Ying’s team created nanofiber patches using polyvinyl alcohol and chitosan, two natural polymers, via single-axis electrospinning and loaded them with various herbal extracts (*Centella asiatica*, purslane, and houttuynia cordata). The drug loading rate was as high as 89.5% to 97.9%, and they maintained high activity in the fibers. The study included 10 patients with varying degrees of acne, and after 14 h of treatment, 8 patients showed significant improvement in their acne lesions [147].

##### Therapeutic Drugs

Some acne treatment medications face challenges such as burst release and low solubility during use, but researchers have found that electrospinning technology offers a solution. To address the issues of poor water solubility, oxidation, light degradation, and thermal degradation of isotretinoin, researchers loaded isotretinoin into polycaprolactone nanofibers using single-axis electrospinning, creating a smooth fiber layer without drug crystals. The nanofibers significantly increased the loading capacity of isotretinoin and effectively achieved a sustained release of isotretinoin to the skin’s surface. Specific experiments showed that isotretinoin exhibited a slow release for up to 4 days, demonstrating that electrospun nanofibers can effectively prolong the release time of isotretinoin, making them potential candidates for acne treatment patches [148]. Similarly, another study showed that electrospinning provides a sustained release effect for medications by encapsulating bee venom peptides in chitosan/polyethylene oxide (PEO) composite nanofibers. The bee venom peptides were released slowly over time, with a release rate of 89.65 ± 2% after 72 h [149] (Table 2). Niacinamide, another acne treatment drug apart from isotretinoin and bee venom peptides, also demonstrated excellent controlled release effects when encapsulated in nanofibers made from a hydroxyethyl cellulose (HEC) and polyvinyl alcohol (PVA) blend.

##### Antibacterial Nanoparticles

Microfluidic methods can prepare drugs in forms more conducive to skin penetration. However, when nanocrystals or particles exist alone, their poor solubility becomes a new issue. Electrospun nanofibers complement this with their high porosity. One study successfully adsorbed resveratrol nanocrystals into the voids of nanofibers, significantly improving solubility and exhibiting anti-inflammatory and antibacterial effects against acne [150]. Zinc oxide (ZnO) particles can destroy bacterial cell membranes and intracellular metabolic processes by releasing a large number of zinc ions, producing hydroxyl radicals and superoxide anions through photocatalytic reactions, then oxidizing key cellular macromolecules to exert antimicrobial effects [151]. A study embedded ZnO particles into the pores of polyvinyl alcohol (PVA) nanofibers, and cross-linked them with citric acid through thermal treatment, enhancing the encapsulation rate of the particles. This patch not only ensures the antibacterial effect of ZnO but also prevents its absorption by the skin, thereby avoiding toxicity, showing good prospects for use in facial masks [152].

**Table 2:** Summary of Electrospinning Applications in Acne Treatment Research.

Electrospinning Condition	Polymer	Solvent	Loading Drug	Outcome			Reference
				Diameter (nm)	Drug Loading Percentage/%	Antibacterial Activity	
Tip to collector distance: 15 cm, solution flow rate: 1.0 mL/h, applied voltage: 25 KV	PVA/10% (*w*/*v*)	Water, Ethanol	Quercetin, Essential oils (tea tree oil, neem oil)	354.95 nm (PVA 10 mL, QC 1 mg, neem oil 1μL, tea tree oil 1 μL). 313.08 nm (PVA 10 mL, QC 2 mg, neem oil 2 μL, tea tree oil 2 μL).	96% ± 0.006	Inhibition zone: 18 ± 0.01 mm	[144]
Tip to collector distance: 15 cm, needle diameter: 1mm, applied voltage: 15 KV	gelatin/30% (*w*/*v*)	Acetic acid	Essential oils from Mentha piperita and Lavandula angustifolia	Gelatin 2% EO: 476.6 ± 51.2 nm. Gelatin 20% EO: 402.6 ± 37.7 nm		MIC: 6.3–9.4 μL/mL. MBC: 9.4–25.0 μL/mL	[145]
Tip to collector distance: 15 cm, solution flow rate: 0.5 mL/h, needle diameter: 0.4mm, applied voltage: 30 KV	PCL (Mn = 80,000)	Chloroform, Methanol	Peppermint oil			MIC: 50–100 μg/mL. MBC: 75–125 μg/mL.	[146]
Tip to collector distance: 15 cm, solution flow rate: 0.1–0.5 mL/h, needle diameter: 1.2 mm, applied voltage: 20 KV	PVA (10% *w*/*v*), chitosan 3.0% *w*/*v*)	Acetic acid	Spray-dried herbal aqueous extracts of *C. asiatica*, *P. oleracea* and *H. cordata*	PVA/CS: 166.10 ± 25.54; PVA/CS/ME3: 159.16 ± 32.38; PVA/CS/ME6: 159.42 ± 43.58; PVA/CS/ME9: 159.52 ± 43.97		*P. acnes* (inhibition zone diameter ≥ 20 mm); *E. coli* (inhibition zone diameter of 16–20 mm); *S. aureus* (inhibition zone diameter of 14–16 mm)	[147]
Tip to collector distance: 16 cm, solution flow rate: 1 mL/h, applied voltage: 19 KV	PCL (10% *w*/*v*)	Dimethylformamide	Tretinoin	PCL (10% *w*/*v*), Tretinoin (0.5% *w*/*v*): 64.67 ± 15.78; PCL (10% *w*/*v*), Tretinoin (1% *w*/*v*): 66.81 ± 15.68 PCL (10% *w*/*v*), Tretinoin (0% *w*/*v*): 80.7 ± 12.67	PCL (10% *w/v*), Tretinoin (0.5% *w*/*v*): 99%; PCL (10% *w*/*v*), Tretinoin (0.5% *w*/*v*): 89%	*S. aureus* ATCC^®^25,923: inhibition zone: 31 mm *S. aureus* ATCC^®^29,213: inhibition zone: 32 mm	[148]
Tip to collector distance: 20 cm, solution flow rate: 0.5 mL/h, needle diameter: 0.9mm, applied voltage: 9 KV	chitosan (2.5% *w*/*v*), PEO (2.5% *w*/*v)*	Acetic acid	Melittin	Ch/Mel0.001%: 550 nm. Ch/Mel0.003%: 600 nm	Ch/Mel0.001%: 74.61 ± 2.5%. Ch/Mel0.003%: 86.74 ± 1%	Ch/Mel 0.003%: 98.02 ± 3.53. free melittin 0.003% in PBS: 97.86% ± 1.84. Ch/Mel 0.001%: 53.9 ± 0.54	[149]
Tip to collector distance: 15 cm, solution flow rate: 0.8–1.0 mL/h, needle diameter: 0.5mm, applied voltage: 17–20 KV	PVA (10% *w*/*v*)	Water	ZnO nanoparticles	the ZnO content increased from 0 wt.% to 7 wt.%: 360 ± 51, 301 ± 60, 300 ± 53, and 325 ± 48 nm, respectively		PVA/ZnO 4%: *S. aureus*: inhibition zone 1.5 mm. PVA/ZnO 7%: *S. aureus*: inhibition zone 1.5 mm, *C. acnes*: inhibition zone 2.25 mm	[150]
Tip to collector distance: 15 cm, solution flow rate: 3 mL/h, applied voltage: 13 KV	PCL	HFIP	Trans-resveratrol	nanocrystals 0.2 mg/cm^2^: 1457 ± 648, nanocrystals 1 mg/cm^2^: 1506 ± 527	nanocrystals 0.2 mg/cm^2^: 89.32%, nanocrystals 0.2 mg/cm^2^: 71.73%	nanocrystals 0.2 mg/cm^2^: *C. acnes* inhibition zone 1.3 ± 0.02 cm, nanocrystals 1 mg/cm^2^: *C. acnes* inhibition zone 1.6 ± 0.1 cm	[152]

MIC: Minimal Inhibition Concentration, MBC: Minimal Bactericidal Concentration.

#### 3.2.3. Electrospinning for Acne: Clinical Progress and Commercial Products

As of now, research on the use of electrospun nanofibers in acne treatment remains in its early stages, with most studies limited to animal experiments or small-scale preclinical trials. Few have advanced to large-scale clinical trials. One exploratory small-sample clinical study applied PVA electrospun nanofiber patches to patients with mild to moderate acne, incorporating tea tree oil and the anti-inflammatory compound quercetin. Preliminary results showed reductions in skin lesions, alleviation of redness and swelling, and good patient tolerability. On average, inflammatory papules were reduced by approximately 61.2%, non-inflammatory lesions (comedones) by about 14.7%, and the total acne lesion count by around 52.9% [144]. Although this small-scale clinical trial yielded promising preliminary results, its open-label, uncontrolled design limits its evidentiary strength. Currently, there is still a lack of mature, acne-targeted commercial nanofiber products. Among the few available is a product from the Czech brand nanoBeauty/nanoSPACE. Compared to traditional sheet masks or gel masks, this fast-dissolving nanofiber mask demonstrates significant advantages in skin absorption efficiency, stability of active ingredients, and user convenience, offering a scientific, safe, and rapid skincare option for acne-prone individuals. In summary, electrospun nanofibers still hold vast potential in the field of acne treatment, but substantial research and developments are needed before clinical application and translation can be fully realized.

## 4. Prospects of Mesenchymal Stem Cell and Exosome-Loaded Electrospun Fibers for Acne Treatment

Electrospinning technology has made significant progress and has wide applications in skin tissue engineering. By adjusting the porosity and specific surface area during the preparation of nanofibers, their biocompatibility can be enhanced, making the nanofiber structure more similar to the natural extracellular matrix (ECM), and allowing for extensive cell attachment and proliferation. In addition to the drugs and plant extracts mentioned earlier, live cells or other biomolecules can also be used as alternative loading materials for nanofibers. For instance, small molecules (antibiotics, amino acids) and biological macromolecules (proteins, nucleic acids) can be incorporated into nanofibers [153].

Since acne is a multifactorial, chronic inflammatory skin disease rather than being caused solely by *Cutibacterium acnes*, the effects of loading plant essential oils, natural extracts, or metal nanoparticles are limited due to their single mode of action and narrow targeting scope. These agents can inhibit bacterial growth to some extent but are insufficient in reversing abnormal sebum secretion, regulating inflammatory cytokine release, or repairing the damaged skin barrier. Moreover, severe acne often involves damage to the dermis, and the aforementioned agents are inadequate in promoting fibroblast regeneration, suppressing the abnormal activation of scar-forming fibroblasts, or facilitating the orderly deposition of collagen. Therefore, combining these agents with electrospun nanofibers is not the optimal strategy for treating acne. However, the emergence of mesenchymal stem cells and their exosomes offers promising potential to overcome these limitations, and we will further discuss their unique therapeutic functions that conventional agents lack. Recent studies combining electrospinning with stem cells or exosomes have shown promising results in wound healing [154,155,156], bone or cartilage regeneration [157,158,159], and nerve regeneration [160,161]. However, there is currently no research on the application of electrospinning combined with stem cells or exosomes in acne treatment. We hypothesize that if mesenchymal stem cells or exosomes, which have tissue repair, anti-inflammatory, and anti-scar effects, are loaded into nanofibers and made into patches for application to acne lesions, this approach may achieve ideal results by reducing inflammation, repairing inflammation-induced skin damage, and inhibiting scar or hyperpigmentation formation.

### 4.1. Nanofibers Combined with Mesenchymal Stem Cells

Stem cells are mainly classified into three types: adult stem cells, embryonic stem cells, and induced pluripotent stem cells [162]. They possess powerful differentiation potential and are considered promising candidates in tissue regeneration engineering, with the ability to self-renew for extended periods and differentiate into any cell type [163]. Mesenchymal stem cells are a type of cell that can differentiate into various types of adult cells. However, issues such as stability, heterogeneity, immunogenicity, differentiation, and migration capabilities remain challenges that need to be addressed for the clinical application of mesenchymal stem cell therapies [164]. The surface receptors of mesenchymal stem cells can target chemokines produced at the wound site, guiding the stem cells to migrate to areas requiring repair [165]. Under the stimulation of inflammatory factors, these cells release various growth factors, such as vascular endothelial growth factor, transforming growth factor β1, keratinocyte growth factor, and epidermal growth factor, which can enhance angiogenesis at the wound site through paracrine effects [166]. A previous study engineered human adipose-derived mesenchymal stem cells to overexpress CXCR4 on their membrane surface, allowing them to interact with stromal cell-derived factor-1 (SDF-1 or CXCL12) at the site of ischemia in mice, promoting the homing of the carrier to the injury site and regulating angiogenesis and hematopoiesis [167]. Growing consensus indicates that mesenchymal stem cells promote wound healing and skin regeneration through anti-inflammatory effects, modulation of the immune microenvironment [168], and enhancement of angiogenesis [169].

In recent years, preclinical studies have emerged to utilize mesenchymal stem cells loaded into nanofibers to exert their anti-inflammatory and tissue repair functions. A study has developed an electrospinning in situ cell delivery system that promotes local tissue healing by directly depositing gelatin fibers and mesenchymal stem cells onto the wound site, effectively overcoming many challenges such as lack of targeting, high cell loss rate, and poor therapeutic effect during stem cell delivery [170]. Another study integrating electrospinning technology with photofabrication techniques developed a gelatine nanofiber network with random modifications that can regulate the paracrine effects of adipose-derived mesenchymal stem cells. This network was shown to influence the migration of fibroblasts and endothelial cells in vitro and accelerate wound healing in a rat model in vivo. It increased the thickness of newly formed epidermis and promoted the formation of type I collagen in the dermis [171].

Inspired by this, we can use nanofibers to induce paracrine effects by loading mesenchymal stem cells onto them. This approach could help stimulate the surrounding tissue at acne lesions to produce a certain amount of collagen, thereby alleviating and supplementing the excessive degradation of collagen or extracellular matrix caused by MMPs during inflammation. This strategy may significantly reduce the formation of atrophic scars during the acne repair process. However, nanofibers still have limitations in efficiently loading mesenchymal stem cells and ensuring their activity. For enhancing the adhesion and proliferation of stem cells on nanofibers, some hydrophilic materials are preferred, and it is necessary to avoid using electrospinning materials with hydrophobic surfaces such as PCL and PLA. In order to better promote the migration and growth of mesenchymal stem cells into fibers, adjustments need to be made to the size, mechanical strength, and degradation of nanofibers. In contrast, using nanofibers to directly load extracellular vesicles from mesenchymal stem cells has more advantages. Extracellular vesicles have lower immunogenicity, stronger standardization, and clearer regulatory strategies than mesenchymal stem cells, making them more controllable in production, storage, and transportation. Therefore, many researchers have begun to explore the application of the combination of nanofibers and mesenchymal stem cell extracellular vesicles.

### 4.2. Nanofibers Loaded with Mesenchymal Stem Cell-Derived Exosomes

Immune modulation systems based on a background of biomaterials have emerged in recent years, guiding the direction of regenerative medicine. Fixing exosomes derived from mesenchymal stem cells onto fibrous polymer meshes can create a new and promising acellular regenerative system [172], which exerts immune regulation and repair functions through the delivery of exosome signals. Allowing exosomes to maintain a long term is the advantage of electrospun nanofibers as carriers, preventing exosomes’ functionality from being compromised in harsh injury environments [173]. Additionally, electrospun fibers strongly mimic the extracellular matrix in terms of fiber morphology and mechanical properties, which is a unique feature not found in other scaffold materials [174].

#### 4.2.1. Anti-Inflammatory and Tissue Repair Functions of Exosomes

Exosomes are extracellular vesicles with diameters ranging from 40 to 160 nm, playing crucial roles in cell-to-cell communication and mediating intracellular signaling [175,176]. Exosomes contain proteins, nucleic acids (DNA, RNA, miRNA, long non-coding RNA), among other substances, and their composition is largely influenced by their originating cells [177]. Due to their low immunogenicity and high biocompatibility, exosomes are widely used in research for various diseases [178]. However, they also have some limitations that have not yet been fully overcome, such as instability, poor targeting, low retention rates, and susceptibility to uptake by neighboring cells [179]. The strategy of combining electrospinning with mesenchymal stem cell-derived exosomes has opened up promising prospects in fields such as cardiac repair [180], wound healing [154,181], diabetic peripheral neuropathy [182], periodontal regeneration [183], and cardiovascular tissue regeneration.

Exosomes have been shown to regulate macrophage polarization towards the M2 phenotype, reducing the release of pro-inflammatory factors while increasing the expression of anti-inflammatory factors, thus enhancing angiogenesis and tissue repair [184]. One study designed a hydrogel loaded with exosomes derived from human umbilical vein endothelial cells under hypoxic conditions, and used it for treating and repairing diabetic wound in mice. The exosomes released by hydrogel effectively promoted angiogenesis and collagen deposition at the wound of diabetes mice and regulated the polarization of macrophages to M2 phenotype [185]. Exosomes derived from M2 macrophages can also induce the transformation of M1 macrophages into M2 macrophages by stimulating the PI3K/AKT pathway, thereby reasonably regulating macrophage subtype polarization and avoiding excessive inflammatory responses that inhibit fracture healing [186]. Another study that combined ECM like hydrogel with bone marrow mesenchymal stem cell (BMSC) exosomes in the rat distal femur drilling growth plate injury model also showed that after phagocytosis of BMSC exosomes, macrophages polarized toward M2 phenotype under immune regulation, the expression of Arg-1 and IL-10 was up-regulated, and the expression levels of iNOS and TNF-α were reduced [187]. Thus, the anti-inflammatory and immunomodulatory effects of mesenchymal stem cell-derived exosomes are likely to be applied in acne treatment. Moderating inflammation can help prevent excessive damage to skin cells, which is crucial for inhibiting the formation of atrophic scars, providing strong theoretical and experimental support for using electrospinning to effectively intervene in acne.

#### 4.2.2. miRNA-Mediated Inhibition of Scar Formation Pathways in Exosomes

Exosomes are rich in a variety of functionally diverse miRNAs, and many studies have indicated that certain miRNAs are important regulators of fibrosis and are associated with scar formation [188,189]. A study compared the expression of miRNAs in hypertrophic scar skin and normal skin tissue samples through miRNA microarray analysis, and found that 74 miRNAs were upregulated and 28 miRNAs were downregulated [190]. Experimental evidence suggests that miR-182-5p can inhibit fibroblast proliferation and migration via the Smad4 pathway, thereby suppressing hypertrophic scar formation [191]. Another study examined the effect of miR-138-5p on pathological scarring, revealing that it can reduce fibroblast proliferation, migration, and protein expression by targeting SIRT1, thus alleviating pathological scar formation [192]. Engineering methods can enhance the content of scar-inhibiting miRNAs in exosomes, which helps to achieve a stronger anti-scar effect. In addition, we summarized several studies on the inhibition of keloid fibroblast activity and proliferation by microRNAs (miRNAs), highlighting the specific signaling pathways involved. Although the targeted proteins vary, their ultimate goal is to promote apoptosis of keloid fibroblasts and thereby inhibit scar formation, which provides both theoretical and practical support for using electrospun nanofibers loaded with engineered mesenchymal stem cell-derived exosomes overexpressing miRNAs to inhibit scar formation [188,193,194,195,196,197,198] (Table 3).

From the above discussion, it is evident that electrospun nanofibers loaded with mesenchymal stem cell-derived exosomes have shown preliminary effects only in preclinical studies involving cell cultures and chronic wound models in animals, demonstrating their potential for repair and anti-inflammatory functions. However, due to the complexity of ethical approval processes, clinical data in this area remain limited and can only provide partial support for our proposed approach. Thus, we can infer that loading miRNAs, which inhibit scar formation pathways, into electrospun nanofibers with extracellular matrix characteristics and applying them to acne-affected areas can help maintain the activity of exosomes on the fibers. Additionally, this approach can promote the release of exosomes from the fiber membrane and facilitate transdermal absorption, thereby effectively inhibiting scar formation at the molecular level.

## 5. Challenges and Future

Although electrospun nanofibers combined with mesenchymal stem cells and their exosomes hold strong theoretical support and promising prospects in the scar-free treatment of acne, several key challenges remain unresolved. In this section, we present a forward-looking and critical perspective from the following aspects: limitations in the use of mesenchymal stem cell-derived exosomes and miRNAs, strategies to enhance the loading capacity and bioactivity of exosomes on electrospun nanofibers, translational challenges and standardized clinical regulatory protocols for bioactive compound-loaded skin patches, and future directions for the personalized design of nanofiber-based skin patches. Our aim is to provide valuable insights and guidance for future research in this field.

### 5.1. Limitations of the Use of Mesenchymal Stem Cell Extracellular Vesicles and miRNAs

(a) Differences in source and biological heterogeneity: MSCs can be derived from various tissues, including bone marrow, adipose tissue, umbilical cord, placenta, and dental pulp, which leads to considerable source-related differences. These differences directly result in significant variations in the content, composition, and functions of their derived exosomes. In addition, exosomal heterogeneity arises from multiple factors. The donor’s age, sex, and health status can influence the phenotype and secretory profile of MSCs. Culture conditions, such as serum type, oxygen concentration, and passage number, also affect the quality of exosomes and their miRNA content. Furthermore, exosomes obtained using different isolation methods may vary in size, purity, and functionality. Therefore, strategies such as standardizing cell sources, optimizing culture conditions, unifying exosome isolation protocols, and screening for key miRNAs can help reduce the heterogeneity of MSC-derived exosomes and improve their standardization.

(b) Low expression levels and off-target effects: Due to limitations in gene transcriptional regulation and processing efficiency, as well as the inherently short half-life and susceptibility to degradation of miRNAs, the endogenous expression levels of miRNAs in cells or exosomes are generally low. This often leads to insufficient regulation of target genes and compromises therapeutic efficacy. In recent years, researchers have employed various strategies to increase miRNA expression levels. These include enhancing miRNA expression in mesenchymal stem cells via transgenic techniques or viral vectors to improve exosomal loading efficiency, and designing protective carriers for exosomes to prevent rapid degradation, thereby increasing the concentration of miRNAs reaching target tissues. Additionally, the combined use of multiple miRNAs to exert synergistic effects can help overcome the limitations of single miRNA efficiency. However, the off-target effects of miRNAs—where they unintentionally regulate non-target genes—can disrupt normal cellular gene networks and potentially cause adverse effects or toxicity, highlighting the need for effective preventive strategies. Feasible approaches include optimizing miRNA sequences, improving targeting techniques, controlling miRNA dosage, and predicting and screening for potential off-target genes.

### 5.2. The Challenge of Efficiently Loading Extracellular Vesicles onto Electrospun Nanofibers and Maintaining Their Activity

There are several challenges associated with the efficient loading of mesenchymal stem cell-derived exosomes into electrospun nanofibers. The complex structure and negatively charged nature of exosomes make it difficult for them to disperse uniformly in traditional polymer solutions. Additionally, the hydrophobicity of nanofibers often results in poor exosome loading affinity. During the electrospinning process, factors such as high voltage, high-speed centrifugation, and solvent evaporation can pose risks to the integrity of the exosomal membrane and the stability of active components such as proteins and nucleic acids. Moreover, direct loading of exosomes is often accompanied by a “burst release” phenomenon, where a large amount of exosomes is released in a short period, making it difficult to achieve sustained and controlled therapeutic effects. Finally, the storage of exosome-loaded nanofibers requires stringent conditions, and there is currently a lack of standardized preservation strategies and stability evaluation systems. These issues highlight the need for further research to overcome technical barriers and improve both the loading efficiency and the preservation of exosomal bioactivity within nanofibers.

### 5.3. Translational Safety and Standardized Clinical Regulatory Protocols for Bioactive Agent-Loaded Patches

(a) Translational safety evaluation: The main components of bioactive patches include active ingredients—such as cytokines, nucleic acids, exosomes, proteins, and peptides—as well as the carrier patch. The biosafety of both components is one of the critical prerequisites for clinical translation. Currently, national regulations have established certain guidelines for the biocompatibility evaluation of biomedical devices. The Technical Guidelines for Cosmetic Safety (2021 edition) stipulate that active substances applied to the skin must undergo toxicological assessments, including tests for acute toxicity, skin irritation, sensitization, phototoxicity, and genotoxicity. There are also mandatory requirements for disclosing the source, purity, structure, and stability of active components. When necessary, preclinical studies are also required. These regulatory measures undoubtedly play an important role in promoting further clinical translation.

(b) Regulatory pathway for clinical translation: In China, the National Medical Products Administration (NMPA) serves as the primary regulatory authority and has established detailed product classifications for bioactive patches. If the active substances in the patch are small-molecule drugs or biologics (such as proteins, peptides, or nucleic acids), they are regulated as pharmaceuticals. Products containing cells or cell-derived components—such as exosomes or vaccines—are managed as biological products. Carriers without bioactive substances are regulated under medical device regulations. Prior to full clinical translation, clinical trial approval is essential, which includes submitting a clinical trial application, conducting Phase I–III clinical trials for drugs, and performing rigorous immunogenicity testing.

(c) Establish standardized strategies: Establishing a comprehensive and systematic standardized strategy for bioactive substance-loaded patches is a long-term goal that requires sustained effort. This can be approached from multiple aspects, including manufacturing processes, functional evaluation, and quality control indicators. A reproducible batch production process should be developed to standardize patch structure, dimensions, and the dosage of loaded bioactive substances. Consistent methods and equipment should be used to standardize the release profiles of active ingredients, and various in vitro cell experiments should be employed to validate the bioactivity of the loaded substances. Consistency in animal models should be ensured before initiating in vivo studies. Additionally, quality control indicators may include the content and distribution of active substances, sterility, and retention rate of active components.

### 5.4. Future Directions: Intelligent Integration and Stimulus Responsiveness for the Design of Personalized Patches

(a) Personalized design of shape and structure: With the deepening advancement of precision medicine strategies, paying attention to and addressing personalized needs has become key to achieving effective treatment. Traditional acne therapies often fail to fully consider individual differences among patients, such as skin pH, sebum secretion levels, lesion locations, and disease severity. This oversight may lead to reduced therapeutic efficacy, poor patient experience, and even potential safety risks. Electrospun nanofiber patches offer flexible shape and structural design capabilities, enabling the personalized customization of patches based on lesion areas such as the nose, forehead, jawline, and cheeks through techniques like 3D modeling and thermoforming, thereby enhancing adhesion to the affected sites. Besides fit and adhesion, easy removability is another outstanding feature of superior skin patches. Incorporating segmented edges with polygonal or petal-like shapes can not only improve adhesion but also reduce the pulling sensation on lesion sites and surrounding tissues during removal. At the structural level, multilayer designs can be implemented by dividing the overall patch into three layers, each assigned specific functions—for example, the outer layer for waterproofing, contamination protection, and antioxidation; the middle layer for loading therapeutic drugs; and the inner layer for absorbing tissue exudate, allowing gas permeability, and maintaining moisture—providing patients with a better treatment experience.

(b) Functional integrated design: Building on personalized customization of shape and structure, multiple functional integrated modules can be incorporated, including sensors that monitor indicators reflecting acne progression such as pH, temperature, and inflammatory factor concentrations at the lesion site, or visual feedback systems that indicate skin status through color changes or fluorescence responses. These features enable real-time monitoring of the patient’s skin condition and dynamic adjustment of treatment intensity as needed. To regulate the release of active substances and achieve long-term therapeutic effects, nanofiber skin patches can be engineered into endogenous or exogenous stimulus-responsive systems. For example, the release of antibacterial drugs, proteins, peptides, and antioxidants can be controlled based on local pH, specific enzymes, reactive oxygen species (ROS), or temperature at the lesion site. Exogenous stimuli include light, electrical, magnetic, and ultrasound triggers, which can induce targeted cell migration and promote drug penetration into deeper tissues.

## 6. Conclusions

To date, researchers have not yet identified a method that can completely cure acne and prevent its recurrence. From the discussion of the four main mechanisms of acne pathogenesis presented in this paper, it is evident that there is a certain interconnection and temporal sequence among them. Current treatments and research primarily target the first three mechanisms: increased sebaceous secretion, excessive follicular keratinization, and microbial imbalance. However, the inflammatory response induced by acne is also a crucial phase that requires attention and investigation, as it often occurs in the later stages of acne and is closely related to the formation of scars and pigmentation. We hypothesize that combining early-stage methods to inhibit excessive sebaceous secretion and antimicrobial treatments with late-stage anti-inflammatory approaches could significantly mitigate the post-acne sequelae, such as scars and pigmentation, that affect facial esthetics.

The diversity of electrospun nanofiber structures determines their rich functionality. Their specific morphology allows them to simultaneously offer excellent skin adhesion, breathability, moisture absorption, and MSC. Additionally, by adjusting the raw materials or structure, strong mechanical strength and elasticity can be achieved—fundamental qualities for effective and user-friendly acne dressings. Under this premise, incorporating drugs with antibacterial, anti-inflammatory, and repair-promoting functions can significantly enhance drug utilization and release performance, reducing the need for repeated applications. From a cellular perspective, high mechanical elasticity helps suppress the sustained activation or excessive differentiation of fibroblasts into myofibroblasts, guiding the dermal layer of the skin to repair damaged cells while avoiding hypertrophic scar formation.

Loading MSCs or their exosomes onto electrospun nanofibers is another potential advantage discussed in this review. The porous structure of electrospun nanofibers supports the retention and growth of MSCs, facilitating anti-inflammatory effects, modulating the appropriate differentiation of various cells in the dermal layer, and promoting the regeneration of type III collagen, thus preventing the formation of atrophic scars. Direct loading of exosomes can also inhibit inflammation by affecting macrophage paracrine activity, primarily through the polarization of M0 macrophages to M2 macrophages, which release more anti-inflammatory factors. The miRNAs in exosomes can also block the expression of genes and pathways related to hypertrophic scar formation, demonstrating their significant potential in acne treatment.

Overall, electrospinning technology undoubtedly holds promise for scar-free acne repair. However, the current challenge is further clinical exploration. Although some studies have shown notable results in animal experiments, key clinical outcomes are still lacking. Additionally, research on the use of exosomes in acne treatment is insufficient, and whether their anti-inflammatory and anti-scar effects can be truly realized in clinical settings remains unknown, necessitating further active exploration by researchers.

## Figures and Tables

**Table 1:** Summary of Clinical Studies on Treatment Effects of Different Types of Lasers Combined with Other Methods for Treating Acne Scars.

Number	Intervention Method/Corresponding Sample Size (Number of Samples)			Cycle	Efficacy Assessment Criteria		Effectiveness Rate (%)	Reference
	Group 1	Group 2	Group 3					
1	Microneedle combined ALA-PDT/16	CO2 fractional laser combined ALA-PDT/28	Injectable corticosteroids/8	treatment 3 times, interval of 1 month	VSS	≥90% recovery ≥60%, <90% significant effect ≥20%, <60% improvement <20% no effect	Group 1: 93.75% Group 2: 100% Group 3: 100%	[80]
2	CO_2_ fractional laser combined with PRP/39	CO_2_ fractional laser treatment/42		treatment 3 times, interval of 1 month	scar repair area	>90% recovery >60%, <90% significant effect <60%, >30% effective <30% no effect	Group 1: 94.87% Group 2: 78.57%	[81]
3	1064 nm Nd:YAG/33	2940 nm Er:YAG/33		treatment 4 times, interval of 4 weeks	IGA	0: no improvement 1: 1–25%improvement 2: 26–50%improvement 3: 51–75%improvement 4: 76–100%improvement	Group 1: 54.84% Group 2: 74.19%	[82]
4	CO_2_ fractional laser combined with TM0.5%/30	CO_2_ fractional laser treatment/30		treatment 3–4 times, interval of 5 weeks	SQGS	1: <25%improvement 2: 26–50%improvement 3: 51–75%improvement 4: >75%improvement	Group 1: 80.00% Group 2: 56.67%	[83]
5	CO_2_ fractional laser treatment/350	CO_2_ fractional laser combined with PRP/350		treatment 3 times, interval of 1 month	VSS		Group 1: 70.00% Group 2: 81.43%	[84]
6	IPL/random (15 in total)	FxPico combined with IPL/random (15 in total)		treatment 5 times, interval of 4 weeks	VISIA	0: 0–24% improvement 1: 25–49% improvement 2: 50–74% improvement 3: 75–100% improvement	Group 2 observed better scar improvement no significant difference in erythema improvement between the two groups	[85]
7	CO_2_ fractional laser/random (21 in totol)	CO_2_ fractional laser combined with ITN/random (21 in total)		treatment 3 times, interval of 4 weeks	GASGS	0: no effect 1: ≤25% mild improvement 2: 26–50% moderate improvement 3: 51–75% significant improvement 4: >75% complete improvement	Group 1: 7.7 ± 2.9% Group 2: 4.7 ± 2.5%	[86]
8	FTL/27	FEL/27		treatment 3 times, interval of 4–6 weeks	GBS		Group 1: 36.54% Group 2: 35.27%	[87]

Table note: ALA-PDT: 5-aminolevulinic acid-based photodynamic therapy; TM: Timolol; IPL: intense pulsed light; FxPico+IPL: fractional 1064 nm Nd: YAG picosecond laser combined with IPL; ITN: isotretinoin; FTL: fractional non-ablative 1927 nm thulium laser; FEL: d fractional ablative 2940 nm Er: YAG laser; VSS: vancouver scar scale; ECCA: échelle d’évaluation clinique des cicatrices d’acné; IGA: Investigator’s Global Assessment; ASAS: acne scar assessment scale; SQGS: scar quartile grading scale; VISIA: assessing the standardized photography; GASGS: Quantitative Global Acne Scarring Grading System; GBS: Goodman&Baron quantitative global scarring grading system.

**Table 3:** Specific miRNA mediated scar inhibition and its complete mechanism of action.

Specific miRNA	Source Cells	Signaling Pathway/Target Protein	Complete Mechanism of Action	References
miR-203a-3p	Myofibroblasts (MFBs)	PI3K/AKT/mTOR, PIK3CA	ESC-Exos releases miR-203a-3p PIK3CA↓ PI3K/Akt signaling pathway↓ α-SMA, COL1A1, FN↓	[188]
miR-7846-3p	Keloid fibroblasts (KFs)	NRP2/Hedgehog/SHH/SMO/GLI1	ADSC-Exos Delivery miR-7846-3p NRP2↓ VEGF, Hedgehog Signal (SHH/SMO/GLI1)↓ The proliferative ability and angiogenic function of KFs↓	[193]
miR-29a	Human corneal stromal stem cells (hCSSCs)	COL1A1/COL3A1/FN1/SPARC/α-SMA/Smad3	hCSSCs secrete exosomes containing miR-29a COL1A1, COL3A1, FN1, SPARC↓ ECM synthesis↓ α-SMA, TGF-β1/Smad3↓	[194]
miR-30a-5p	Keloid fibroblasts (KFs)	BCL2	TSA treatment miR-30a-5p↑ BCL2 (anti apoptotic protein)↓	[195]
miR-4417	Keloid fibroblasts (KFs)	CyclinD1	miR-4417↑ CyclinD1↓ KFs proliferation and migration↓	[196]
miR-152-3p	Keloid fibroblasts (KFs)	FOXF1	miR-152-3p↑ FOXF1↓ cell proliferation↓ Type I and III collagen, fibronectin↓	[197]
miR-152-5p	Keloid fibroblasts (KFs)	Smad3, p-Erk1/2, p-Akt	miR-152-5p↑ Smad3↑ p-Erk1/2, p-Akt↑ Collagen III↑	[198]

Table note: The arrow represents the upregulation (arrow up) or downregulation (arrow down) of a certain miRNA or protein.

## Data Availability

The datasets used and/or analyzed during the current study are available from the corresponding author upon reasonable request.

## Abbreviations

Abbreviations	Full words	Abbreviations	Full words
ACM	acellular matrix	lncRNA	long non-coding RNAs
ACTH	adrenocorticotrophic hormone	LTA	lipoteichoic acid
AD	adapalene	miRNA	microRNA
ADSC	adipose mesenchymal stem cells	MMPs	matrix metalloproteinase
AgNP	Ag nanoparticles	mTORC1	mammalian target of rapamycin1
ASC	Recombinant Asc Type Amino Acid Transporter	NLRP3	NOD-like receptor pyrin domain-containing 3
Caspase-1	cysteinyl aspartate specific proteinase1	PCL	Polycaprolactone
CMCS	carboxymethyl chitosan	PEEK	poly-ether-ether-ketone
SA	sodium alginate	PLGA	Poly(L-lactide-co-glycolide)
DexMA	mutilated dextran	PLLA	Poly(L-lactic acid)
DHT	dihydrotestosterone	PNG	peptidoglycan
ECM	extracellularmatrix	PPAR-γ	peroxisome proliferator-activated receptor γ
exos	exosomes	Pro-caspase-1	pro cysteinyl aspartate specific proteinase 1
FGFR	fibroblast growth factor receptor	Pro-IL-1β	pro interleukin-1β
FoxO1	forkhead box O1	PRP	platelet rich plasma
GELMA	methacrylate-based hydrogel	PVA	polyvinylalcohol
HA	hyaluronicacid	SCFA	short-chain fatty acids
IGF-1	insulin-like growth factor-1	SREPB-1c	Sterol-regulatory element binding proteins-1c
IL-12	interleukin-12	TAK1	TGF-β-activated kinase 1
IL-1α	interleukin-1α	TGF-β	transforming growth factor-β
IL-1β	interleukin-1β	Th17	T helper cell 17
IL-6	interleukin-6	TNF-α	tumor necrosis factor-α
IL-8	interleukin-8	TRL-2	Toll-like Receptor 2
TRL-4	Toll-like Receptor 4	*C. acnes*	*Cutibacterium acnes*
PET	polyethylene terephthalate	MIC	Minimum inhibitory concentrations
HEC	hydroxyethyl cellulose	BMSC	bone marrow mesenchymal stem cell
MBC	minimum bactericidal concentrations		

## References

[1] Do T., Ma F., Andrade P., Teles R., de Andrade Silva B., Hu C., Espinoza A., Hsu J., Cho C., Kim M. (2022). TREM2 macrophages induced by human lipids drive inflammation in acne lesions. Sci. Immunol..

[2] Asai Y., Baibergenova A., Dutil M., Humphrey S., Hull P., Lynde C., Poulin Y., Shear N., Tan J., Toole J. (2016). Management of acne: Canadian clinical practice guideline. CMAJ Can. Med. Assoc. J. = J. L’association Medicale Can..

[3] Kutlu O., Karadag A.S., Wollina U. (2023). Adult acne versus adolescent acne: A narrative review with a focus on epidemiology to treatment. An. Bras. Dermatol..

[4] Moradi Tuchayi S., Makrantonaki E., Ganceviciene R., Dessinioti C., Feldman S., Zouboulis C. (2015). Acne vulgaris. Nat. Rev. Dis. Primers.

[5] Hay R., Johns N., Williams H., Bolliger I., Dellavalle R., Margolis D., Marks R., Naldi L., Weinstock M., Wulf S. (2014). The global burden of skin disease in 2010: An analysis of the prevalence and impact of skin conditions. J. Investig. Dermatol..

[6] Ramrakha S., Fergusson D., Horwood L., Dalgard F., Ambler A., Kokaua J., Milne B., Poulton R. (2016). Cumulative mental health consequences of acne: 23-year follow-up in a general population birth cohort study. Br. J. Dermatol..

[7] Bernales Salinas A. (2021). Acne vulgaris: Role of the immune system. Int. J. Dermatol..

[8] Peng D.D., Fu M.Y., Wang M.N., Wei Y.Q., Wei X.W. (2022). Targeting TGF-β signal transduction for fibrosis and cancer therapy. Mol. Cancer.

[9] Akhouy G., Aziz K., Gebrati L., El Achaby M., Akgul Y., Yap P.S., Kurniawan T.A., Aziz F. (2023). Recent applications on biopolymers electrospinning: Strategies, challenges and way forwards. Polym.-Plast. Technol. Mater..

[10] Lee J.W., Song K.H. (2023). Fibrous hydrogels by electrospinning: Novel platforms for biomedical applications. J. Tissue Eng..

[11] Si Y.F., Guo C.X., Xu X.Y., Zhang K., Tan R.J., Lau K.T., Hu J.L. (2022). Bioinspired Janus All-Natural Electrospinning Membranes with Directional Water Transport as Ecofriendly Dry Facial Masks. ACS Sustain. Chem. Eng..

[12] Guertler A.L., Rades T., Heinz A. (2023). Electrospun fibers for the treatment of skin diseases. J. Control. Release.

[13] Wang L., Ma J.Y., Guo T., Zhang F.H., Dong A.M., Zhang S.Q., Liu Y.J., Yuan H.P., Leng J.S. (2023). Control of Surface Wrinkles on Shape Memory PLA/PPDO Micro-nanofibers and Their Applications in Drug Release and Anti-scarring. Adv. Fiber Mater..

[14] Gohar S., Iqbal M.A., Mayakrishnan G., Miah M.S., Liang F.H., Kim K.O., Ullah A., Kim I.S. (2025). Therapeutic complex-loaded polyvinyl alcohol nanofibers enriched with lemon juice extract for controlled drug release in acne management. Mater. Chem. Phys..

[15] Hao Y., Lin X., Liu W., Jiang T., Zhang X., Yang S., Huang Y., Lai W., Fu C., Zhang Z. (2025). Development of nanofiber facial mask inspired by the multi-function of dried ginger (Zingiberis Rhizoma) essential oil. Sci. Rep..

[16] Xue Z.X., Liao Y.J., Li Y. (2024). Effects of microenvironment and biological behavior on the paracrine function of stem cells. Genes Dis..

[17] Elhabal S.F., Abdelmonem R., El Nashar R.M., Elrefai M.F.M., Hamdan A.M.E., Safwat N.A., Shoela M.S., Hassan F.E., Rizk A., Kabil S.L. (2024). Enhanced Antibacterial Activity of Clindamycin Using Molecularly Imprinted Polymer Nanoparticles Loaded with Polyurethane Nanofibrous Scaffolds for the Treatment of Acne Vulgaris. Pharmaceutics.

[18] Perera M.P.N., Peiris W.M.D.M., Pathmanathan D., Mallawaarachchi S., Karunathilake I.M. (2018). Relationship between acne vulgaris and cosmetic usage in Sri Lankan urban adolescent females. J. Cosmet. Dermatol..

[19] Conforti C., Agozzino M., Emendato G., Fai A., Fichera F., Marangi G.F., Neagu N., Pellacani G., Persichetti P., Segreto F. (2022). Acne and diet: A review. Int. J. Dermatol..

[20] Suh D., Kwon H. (2015). What’s new in the physiopathology of acne?. Br. J. Dermatol..

[21] Dréno B. (2017). What is new in the pathophysiology of acne, an overview. J. Eur. Acad. Dermatol. Venereol..

[22] Kurokawa I., Layton A.M., Ogawa R. (2021). Updated Treatment for Acne: Targeted Therapy Based on Pathogenesis. Dermatol. Ther..

[23] Dréno B., Dagnelie M.A., Khammari A., Corvec S. (2020). The Skin Microbiome: A New Actor in Inflammatory Acne. Am. J. Clin. Dermatol..

[24] Dagnelie M.-A., Corvec S., Saint-Jean M., Bourdès V., Nguyen J.-M., Khammari A., Dréno B. (2017). Decrease in Diversity of Propionibacterium acnes Phylotypes in Patients with Severe Acne on the Back. Acta Derm.-Venereol..

[25] Dagnelie M.-A., Corvec S., Saint-Jean M., Nguyen J.-M., Khammari A., Dréno B. (2019). Cutibacterium acnes phylotypes diversity loss: A trigger for skin inflammatory process. J. Eur. Acad. Dermatol. Venereol..

[26] Hernandez M., López P., Gaete X., Villarroel C., Cavada G., Avila A., Iñiguez G., Cassorla F. (2017). Hyperandrogenism in adolescent girls: Relationship with the somatotrophic axis. J. Pediatr. Endocrinol. Metab..

[27] Hu T., Wei Z., Ju Q., Chen W. (2021). Sex hormones and acne: State of the art. J. Ger. Soc. Dermatol..

[28] AbdElneam A., Al-Dhubaibi M., Bahaj S., Mohammed G., Atef L. (2023). Apo B-48 gene expression and low-density lipoprotein as a factor for increased insulin resistance and severity of acne. Gene.

[29] Ben-Amitai D., Laron Z. (2011). Effect of insulin-like growth factor-1 deficiency or administration on the occurrence of acne. J. Eur. Acad. Dermatol. Venereol..

[30] Zouboulis C., Dessinioti C., Tsatsou F., Gollnick H. (2017). Anti-acne drugs in phase 1 and 2 clinical trials. Expert Opin. Investig. Drugs.

[31] Cottle D., Kretzschmar K., Schweiger P., Quist S., Gollnick H., Natsuga K., Aoyagi S., Watt F. (2013). c-MYC-induced sebaceous gland differentiation is controlled by an androgen receptor/p53 axis. Cell Rep..

[32] Kumtornrut C., Yamauchi T., Koike S., Aiba S., Yamasaki K. (2019). Androgens modulate keratinocyte differentiation indirectly through enhancing growth factor production from dermal fibroblasts. J. Dermatol. Sci..

[33] Su Z., Zhang Y., Cao J., Sun Y., Cai Y., Zhang B., He L., Zhang Z., Xie J., Meng Q. (2023). Hyaluronic acid-FGF2-derived peptide bioconjugates for suppression of FGFR2 and AR simultaneously as an acne antagonist. J. Nanobiotechnol..

[34] Agamia N., Hussein O., Abdelmaksoud R., Abdalla D., Talaat I., Zaki E., El Tawdy A., Melnik B. (2018). Effect of oral isotretinoin on the nucleo-cytoplasmic distribution of FoxO1 and FoxO3 proteins in sebaceous glands of patients with acne vulgaris. Exp. Dermatol..

[35] Chibaya L., Karim B., Zhang H., Jones S. (2021). Mdm2 phosphorylation by Akt regulates the p53 response to oxidative stress to promote cell proliferation and tumorigenesis. Proc. Natl. Acad. Sci. USA.

[36] Laplante M., Sabatini D. (2013). Regulation of mTORC1 and its impact on gene expression at a glance. J. Cell Sci..

[37] Thiboutot D.M. (2000). The role of follicular hyperkeratinization in acne. J. Dermatol. Treat..

[38] Leyden J. (1995). New understandings of the pathogenesis of acne. J. Am. Acad. Dermatol..

[39] Degitz K., Ochsendorf F. (2008). Acne. Current pathophysiologic considerations. Der Hautarzt Z. Dermatol. Venerol. Verwandte Geb..

[40] OShaughnessy R.F.L., Choudhary I., Harper J.I. (2010). Interleukin-1 alpha blockade prevents hyperkeratosis in an model of lamellar ichthyosis. Hum. Mol. Genet..

[41] Cunliffe W., Holland D., Clark S., Stables G. (2003). Comedogenesis: Some aetiological, clinical and therapeutic strategies. Dermatology.

[42] Abdallah F., Mijouin L., Pichon C. (2017). Skin Immune Landscape: Inside and Outside the Organism. Mediat. Inflamm..

[43] Grice E., Segre J. (2011). The skin microbiome. Nat. Rev. Microbiol..

[44] O’Neill A., Gallo R. (2018). Host-microbiome interactions and recent progress into understanding the biology of acne vulgaris. Microbiome.

[45] Jończyk-Matysiak E., Weber-Dąbrowska B., Żaczek M., Międzybrodzki R., Letkiewicz S., Łusiak-Szelchowska M., Górski A. (2017). Propionibacterium acnesProspects of Phage Application in the Treatment of Acne Caused by. Front. Microbiol..

[46] Fitz-Gibbon S., Tomida S., Chiu B., Nguyen L., Du C., Liu M., Elashoff D., Erfe M., Loncaric A., Kim J. (2013). Propionibacterium acnes strain populations in the human skin microbiome associated with acne. J. Investig. Dermatol..

[47] Barnard E., Shi B., Kang D., Craft N., Li H. (2016). The balance of metagenomic elements shapes the skin microbiome in acne and health. Sci. Rep..

[48] Almoughrabie S., Cau L., Cavagnero K., O’Neill A., Li F., Roso-Mares A., Mainzer C., Closs B., Kolar M., Williams K. (2023). Cutibacterium acnesCommensal induce epidermal lipid synthesis important for skin barrier function. Sci. Adv..

[49] Xu H., Li H. (2019). Acne, the Skin Microbiome, and Antibiotic Treatment. Am. J. Clin. Dermatol..

[50] Dreno B., Martin R., Moyal D., Henley J., Khammari A., Seité S. (2017). Skin microbiome and acne vulgaris: Staphylococcus, a new actor in acne. Exp. Dermatol..

[51] Gannesen A., Zdorovenko E., Botchkova E., Hardouin J., Massier S., Kopitsyn D., Gorbachevskii M., Kadykova A., Shashkov A., Zhurina M. (2019). Composition of the Biofilm Matrix of Cutibacterium acnes Acneic Strain RT5. Front. Microbiol..

[52] Kuehnast T., Cakar F., Weinhäupl T., Pilz A., Selak S., Schmidt M., Rüter C., Schild S. (2018). Comparative analyses of biofilm formation among different Cutibacterium acnes isolates. Int. J. Med. Microbiol..

[53] Szegedi A., Dajnoki Z., Bíró T., Kemény L., Törőcsik D. (2019). Acne: Transient Arrest in the Homeostatic Host-Microbiota Dialog?. Trends Immunol..

[54] Graham G., Farrar M., Cruse-Sawyer J., Holland K., Ingham E. (2004). Proinflammatory cytokine production by human keratinocytes stimulated with Propionibacterium acnes and P. acnes GroEL. Br. J. Dermatol..

[55] Sugisaki H., Yamanaka K., Kakeda M., Kitagawa H., Tanaka K., Watanabe K., Gabazza E., Kurokawa I., Mizutani H. (2009). Increased interferon-gamma, interleukin-12p40 and IL-8 production in Propionibacterium acnes-treated peripheral blood mononuclear cells from patient with acne vulgaris: Host response but not bacterial species is the determinant factor of the disease. J. Dermatol. Sci..

[56] Takeuchi O., Hoshino K., Kawai T., Sanjo H., Takada H., Ogawa T., Takeda K., Akira S. (1999). Differential roles of TLR2 and TLR4 in recognition of gram-negative and gram-positive bacterial cell wall components. Immunity.

[57] Sanford J., Zhang L., Williams M., Gangoiti J., Huang C., Gallo R. (2016). Inhibition of HDAC8 and HDAC9 by microbial short-chain fatty acids breaks immune tolerance of the epidermis to TLR ligands. Sci. Immunol..

[58] Broz P., Dixit V. (2016). Inflammasomes: Mechanism of assembly, regulation and signalling. Nat. Rev. Immunol..

[59] Lu A., Magupalli V., Ruan J., Yin Q., Atianand M., Vos M., Schröder G., Fitzgerald K., Wu H., Egelman E. (2014). Unified polymerization mechanism for the assembly of ASC-dependent inflammasomes. Cell.

[60] Kistowska M., Gehrke S., Jankovic D., Kerl K., Fettelschoss A., Feldmeyer L., Fenini G., Kolios A., Navarini A., Ganceviciene R. (2014). IL-1β drives inflammatory responses to propionibacterium acnes in vitro and in vivo. J. Investig. Dermatol..

[61] Li X., Luo S., Chen X., Li S., Hao L., Yang D. (2022). Adipose-derived stem cells attenuate acne-related inflammation via suppression of NLRP3 inflammasome. Stem Cell Res. Ther..

[62] Mias C., Mengeaud V., Bessou-Touya S., Duplan H. (2023). Recent advances in understanding inflammatory acne: Deciphering the relationship between Cutibacterium acnes and Th17 inflammatory pathway. J. Eur. Acad. Dermatol. Venereol..

[63] Sardana K., Verma G. (2017). Propionibacterium acnes and the Th1/Th17 Axis, Implications in Acne Pathogenesis and Treatment. Indian J. Dermatol..

[64] Huang Y., Yang C., Li T., Zouboulis C., Hsu H. (2015). Cell-free extracts of Propionibacterium acnes stimulate cytokine production through activation of p38 MAPK and Toll-like receptor in SZ95 sebocytes. Life Sci..

[65] Lee H., Jang Y. (2018). Recent Understandings of Biology, Prophylaxis and Treatment Strategies for Hypertrophic Scars and Keloids. Int. J. Mol. Sci..

[66] Yang J., Yoon J., Moon J., Min S., Kwon H., Suh D. (2018). Expression of inflammatory and fibrogenetic markers in acne hypertrophic scar formation: Focusing on role of TGF-β and IGF-1R. Arch. Dermatol. Res..

[67] Sabat R., Grütz G., Warszawska K., Kirsch S., Witte E., Wolk K., Geginat J. (2010). Biology of interleukin-10. Cytokine Growth Factor Rev..

[68] Honardoust D., Varkey M., Marcoux Y., Shankowsky H., Tredget E. (2012). Reduced decorin, fibromodulin, and transforming growth factor-β3 in deep dermis leads to hypertrophic scarring. J. Burn Care Res..

[69] van der Veer W.M., Bloemen M.C.T., Ulrich M.M.W., Molema G., van Zuijlen P.P., Middelkoop E., Niessen F.B. (2009). Potential cellular and molecular causes of hypertrophic scar formation. Burns.

[70] Zaleski-Larsen L.A., Fabi S.G., McGraw T., Taylor M. (2016). Acne Scar Treatment: A Multimodality Approach Tailored to Scar Type. Dermatol. Surg..

[71] Moon J., Yoon J., Yang J., Kwon H., Min S., Suh D. (2019). Atrophic acne scar: A process from altered metabolism of elastic fibres and collagen fibres based on transforming growth factor-β1 signalling. Br. J. Dermatol..

[72] Mohammadi A.H., Seirafianpour F., Khosravi M., Jafarzadeh A., Kashi H.N., Baradaran H., Goodarzi A. (2025). A systematic review of comparative clinical trials on the efficacy, safety, and patient satisfaction of ablative and non-ablative laser therapies for atrophic, hypertrophic, and keloid scars. Lasers Med. Sci..

[73] Elwan N.M., Neinaa Y.M.E., Elkhouly R.M., Dagher R.M., Salaam S.F.A. (2025). Intralesional injection of vitamin D3, platelet rich plasma versus their combination in treatment of keloid: A clinical, radiological and immunohistochemical study. Arch. Dermatol. Res..

[74] Keshk Z.S., Salah M.M., Samy N.A. (2025). Fractional carbon dioxide laser treatment of hypertrophic scar clinical and histopathological evaluation. Lasers Med. Sci..

[75] El-Tayeb N.M., Fattah N.S.A.A., El-Badawy N., El-Samahy M.H. (2025). The effectiveness of fractional laser-assisted photodynamic therapy utilizing methylene blue for the treatment of keloids. Arch. Dermatol. Res..

[76] Guadanhim L.R.S., Gonçalves R.G., Bagatin E. (2016). Observational retrospective study evaluating the effects of oral isotretinoin in keloids and hypertrophic scars. Int. J. Dermatol..

[77] Veitch D., Kravvas G., Al-Niaimi F. (2017). Pulsed Dye Laser Therapy in the Treatment of Warts: A Review of the Literature. Dermatol. Surg..

[78] van Jarwaarde J.A., Wessels R., Nieweg O.E., Wouters M.W.J.M., van der Hage J.A. (2015). CO Laser Treatment for Regional Cutaneous Malignant Melanoma Metastases. Dermatol. Surg..

[79] Baleg S., Bidin N., Suan L., Ahmad M., Krishnan G., Johari A., Hamid A. (2015). The effect of CO_2_ laser treatment on skin tissue. J. Cosmet. Dermatol..

[80] Yan D.M., Zhao H.Y., Li C.X., Xia A.T., Zhang J.J., Zhang S., Yun Q., Li X.X., Huang F., Tian Y. (2022). A clinical study of carbon dioxide lattice laser-assisted or microneedle-assisted 5-aminolevulinic acid-based photodynamic therapy for the treatment of hypertrophic acne scars. Photodermatol. Photoimmunol. Photomed..

[81] Guo R., Xuan W.X., He X., Xu K. (2023). Safety and efficacy of CO dot matrix laser combined with platelet-rich plasma on depressed scar after acne vulgaris and influencing factors of its repair effect: A retrospective analysis. J. Cosmet. Dermatol..

[82] Dai R., Cao Y.Y., Su Y.P., Cai S.Q. (2023). Comparison of 1064-nm Nd:YAG picosecond laser using fractional micro-lens array vs. ablative fractional 2940-nm Er:YAG laser for the treatment of atrophic acne scar in Asians: A 20-week prospective, randomized, split-face, controlled pilot study. Front. Med..

[83] Hawwas A., Mohamed H., Sayedahmed O., Elsaie M. (2023). Topical timolol maleate 0.5% after fractional carbon dioxide laser versus fractional carbon dioxide laser alone in treatment of acne scars: Split face comparative study. Sci. Rep..

[84] Wang Y.Y., Yu W., Zhang J.F., Li J.S. (2022). Effect and Safety Analysis of PRP and Yifu Combined with Ultrapulsed CO Lattice Laser in Patients with Sunken Acne Scar. J. Healthc. Eng..

[85] Feng H., Wu Y.M., Jiang M., Luo X.Q., Yan S.X., Lu Z. (2021). The Efficacy and Safety of Fractional 1064 nm Nd:YAG Picosecond Laser Combined with Intense Pulsed Light in the Treatment of Atrophic Acne Scar: A Split-Face Study. Lasers Surg. Med..

[86] Taleb E., Gallo E.S., Salameh F., Koren A., Shehadeh W., Artzi O. (2023). Fractional ablative CO laser and oral isotretinoin—A prospective randomized controlled split-face trial comparing concurrent versus delayed laser treatment for acne scars. Lasers Surg. Med..

[87] Lu K.N., Cai S.Q. (2022). Efficacy and safety comparison between 1927 nm thulium laser and 2940 nm Er:YAG laser in the treatment of facial atrophic acne scarring: A prospective, simultaneous spilt-face clinical trial. Lasers Med. Sci..

[88] Garg K., Bowlin G. (2011). Electrospinning jets and nanofibrous structures. Biomicrofluidics.

[89] Melendez-Rodriguez B., Torres-Giner S., Lorini L., Valentino F., Sammon C., Cabedo L., Lagaron J. (2020). Valorization of Municipal Biowaste into Electrospun Poly(3-hydroxybutyrate-3-hydroxyvalerate) Biopapers for Food Packaging Applications. ACS Appl. Bio Mater..

[90] Xu S.S., Zhang J., He A.H., Li J.X., Zhang H., Han C.C. (2008). Electrospinning of native cellulose from nonvolatile solvent system. Polymer.

[91] Fadil F., Affandi N.D.N., Misnon M.I., Bonnia N.N., Harun A.M., Alam M.K. (2021). Review on Electrospun Nanofiber-Applied Products. Polymers.

[92] Hu X., Liu S., Zhou G., Huang Y., Xie Z., Jing X. (2014). Electrospinning of polymeric nanofibers for drug delivery applications. J. Control. Release.

[93] Norzain N.A., Lin W.C. (2022). Orientated and diameter-controlled fibrous scaffolds fabricated using the centrifugal electrospinning technique for stimulating the behaviours of fibroblast cells. J. Ind. Text..

[94] Feng W., Zhang Y.S., Shao Y.W., Huang T., Zhang N., Yang J.H., Qi X.D., Wang Y. (2021). Coaxial electrospun membranes with thermal energy storage and shape memory functions for simultaneous thermal/moisture management in personal cooling textiles. Eur. Polym. J..

[95] Nagy Z.K., Balogh A., Démuth B., Pataki H., Vigh T., Szabó B., Molnár K., Schmidt B.T., Horák P., Marosi G. (2015). High speed electrospinning for scaled-up production of amorphous solid dispersion of itraconazole. Int. J. Pharm..

[96] Tomar Y., Pandit N., Priya S., Singhvi G. (2023). Evolving Trends in Nanofibers for Topical Delivery of Therapeutics in Skin Disorders. Acs Omega.

[97] Jain R., Shetty S., Yadav K.S. (2020). Unfolding the electrospinning potential of biopolymers for preparation of nano fi bers. J. Drug Deliv. Sci. Technol..

[98] Chandra N.S., Gorantla S., Priya S., Singhvi G. (2022). Insight on updates in polysaccharides for ocular drug delivery. Carbohydr. Polym..

[99] Rogina A. (2014). Electrospinning process: Versatile preparation method for biodegradable and natural polymers and biocomposite systems applied in tissue engineering and drug delivery. Appl. Surf. Sci..

[100] Huang Y.L., Shi R., Gong M., Zhang J.S., Li W.Y., Song Q.P., Wu C.G., Tian W. (2018). Icariin-loaded electrospun PCL/gelatin sub-microfiber mat for preventing epidural adhesions after laminectomy. Int. J. Nanomed..

[101] Zhao L., Li X.F., Yang L., Sun L.L., Mu S.F., Zong H.B., Li Q., Wang F.Y., Song S., Yang C.Q. (2021). Evaluation of remodeling and regeneration of electrospun PCL/fibrin vascular grafts in vivo. Mater. Sci. Eng. C-Mater. Biol. Appl..

[102] Fang Y., Zhu X.Q., Wang N., Zhang X., Yang D.Z., Nie J., Ma G.P. (2019). Biodegradable core-shell electrospun nanofibers based on PLA and γ-PGA for wound healing. Eur. Polym. J..

[103] Zhao W., Li J.J., Jin K.X., Liu W.L., Qiu X.F., Li C.R. (2016). Fabrication of functional PLGA-based electrospun scaffolds and their applications in biomedical engineering. Mater. Sci. Eng. C-Mater. Biol. Appl..

[104] Lee C.H., Huang S.C., Hung K.C., Cho C.J., Liu S.J. (2022). Enhanced Diabetic Wound Healing Using Electrospun Biocompatible PLGA-Based Saxagliptin Fibrous Membranes. Nanomaterials.

[105] Kucinska-Lipka J., Gubanska I., Janik H., Sienkiewicz M. (2015). Fabrication of polyurethane and polyurethane based composite fibres by the electrospinning technique for soft tissue engineering of cardiovascular system. Mater. Sci. Eng. C-Mater. Biol. Appl..

[106] Malik S., Sundarrajan S., Hussain T., Nazir A., Ramakrishna S. (2021). Fabrication of Highly Oriented Cylindrical Polyacrylonitrile, Poly(lactide-glycolide), Polycaprolactone and Poly(vinyl acetate) Nanofibers for Vascular Graft Applications. Polymers.

[107] Hu M., Li C.W., Li X., Zhou M., Sun J.B., Sheng F.F., Shi S.J., Lu L.C. (2018). Zinc oxide/silver bimetallic nanoencapsulated in PVP/PCL nanofibres for improved antibacterial activity. Artif. Cells Nanomed. Biotechnol..

[108] Bombin A.D.J., Dunne N.J., McCarthy H.O. (2020). Electrospinning of natural polymers for the production of nanofibres for wound healing applications. Mater. Sci. Eng. C-Mater. Biol. Appl..

[109] Movahedi M., Asefnejad A., Rafienia M., Khorasani M.T. (2020). Potential of novel electrospun core-shell structured polyurethane/starch (hyaluronic acid) nanofibers for skin tissue engineering. Int. J. Biol. Macromol..

[110] Rashtchian M., Hivechi A., Bahrami S.H., Milan P.B., Simorgh S. (2020). Fabricating alginate/poly(caprolactone) nanofibers with enhanced bio-mechanical properties via cellulose nanocrystal incorporation. Carbohydr. Polym..

[111] Khamrai M., Banerjee S.L., Paul S., Samanta S., Kundu P.P. (2019). Curcumin entrapped gelatin/ionically modified bacterial cellulose based self-healable hydrogel film: An eco-friendly sustainable synthesis method of wound healing patch. Int. J. Biol. Macromol..

[112] Ndlovu S.P., Ngece K., Alven S., Aderibigbe B.A. (2021). Gelatin-Based Hybrid Scaffolds: Promising Wound Dressings. Polymers.

[113] Rachmiel D., Anconina I., Rudnick-Glick S., Halperin-Sternfeld M., Adler-Abramovich L., Sitt A. (2021). Hyaluronic Acid and a Short Peptide Improve the Performance of a PCL Electrospun Fibrous Scaffold Designed for Bone Tissue Engineering Applications. Int. J. Mol. Sci..

[114] Choi C., Yun E., Song M., Kim J., Son J.S., Cha C. (2024). Multiscale Control of Nanofiber-Composite Hydrogel for Complex 3D Cell Culture by Extracellular Matrix Composition and Nanofiber Alignment. Biomater. Res..

[115] Wang L., Li T., Wang Z., Hou J., Liu S., Yang Q., Yu L., Guo W., Wang Y., Guo B. (2022). Injectable remote magnetic nanofiber/hydrogel multiscale scaffold for functional anisotropic skeletal muscle regeneration. Biomaterials.

[116] Majidi S.S., Slemming-Adamsen P., Hanif M., Zhang Z., Wang Z., Chen M. (2018). Wet electrospun alginate/gelatin hydrogel nanofibers for 3D cell culture. Int. J. Biol. Macromol..

[117] Bosworth L.A., Turner L.A., Cartmell S.H. (2013). State of the art composites comprising electrospun fibres coupled with hydrogels: A review. Nanomed. -Nanotechnol. Biol. Med..

[118] Chen Y.J., Hao Y., Mensah A., Lv P.F., Wei Q.F. (2022). Bio-inspired hydrogels with fibrous structure: A review on design and biomedical applications. Biomater. Adv..

[119] Martin A., Nyman J.N., Reinholdt R., Cai J., Schaedel A.L., van der Plas M.J.A., Malmsten M., Rades T., Heinz A. (2022). In Situ Transformation of Electrospun Nanofibers into Nanofiber-Reinforced Hydrogels. Nanomaterials.

[120] Islam M.S., Molley T.G., Hung T.T., Sathish C.I., Putra V.D.L., Jalandhra G.K., Ireland J., Li Y.C., Yi J.B., Kruzic J.J. (2023). Magnetic Nanofibrous Hydrogels for Dynamic Control of Stem Cell Differentiation. ACS Appl. Mater. Interfaces.

[121] Kim D., Kim Y.H., Lee G., Lee E.C., Bhang S.H., Lee K. (2025). Multidimensional nanofibrous hydrogels integrated triculture system for advanced myocardial regeneration. Biofabrication.

[122] Jang W.G., Jeon K.S., Byun H.S. (2013). The preparation of porous polyamide-imide nanofiber membrane by using electrospinning for MF application. Desalination Water Treat..

[123] Yu J., Qiu Y.J., Zha X.X., Yu M., Yu J.L., Rafique J., Yin J. (2008). Production of aligned helical polymer nanofibers by electrospinning. Eur. Polym. J..

[124] Li Y.F., Ma X.M., Deng N.P., Kang W.M., Zhao H.H., Li Z.J., Cheng B.W. (2017). Electrospun SiO/PMIA. Nanofiber Membranes with Higher Ionic Conductivity for High Temperature Resistance Lithium-ion Batteries. Fibers Polym..

[125] Jeckson T.A., Neo Y.P., Sisinthy S.P., Gorain B. (2021). Delivery of Therapeutics from Layer-by-Layer Electrospun Nanofiber Matrix for Wound Healing: An Update. J. Pharm. Sci..

[126] Ghasemiyeh P., Mohammadi-Samani S., Noorizadeh K., Zadmehr O., Rasekh S., Mohammadi-Samani S., Dehghan D. (2022). Novel topical drug delivery systems in acne management: Molecular mechanisms and role of targeted delivery systems for better therapeutic outcomes. J. Drug Deliv. Sci. Technol..

[127] Dagnelie M., Poinas A., Dréno B. (2022). What is new in adult acne for the last 2 years: Focus on acne pathophysiology and treatments. Int. J. Dermatol..

[128] Priya S., Desai V.M., Singhvi G. (2023). Surface Modification of Lipid-Based Nanocarriers: A Potential Approach to Enhance Targeted Drug Delivery. Acs Omega.

[129] Shahriar S., Mondal J., Hasan M., Revuri V., Lee D., Lee Y. (2019). Electrospinning Nanofibers for Therapeutics Delivery. Nanomaterials.

[130] Christodoulou E., Chondromatidou A., Bikiaris N.D., Balla E., Vlachou M., Barmpalexis P., Bikiaris D.N. (2025). PLA-Based Electrospun Nanofibrous Mats Towards Application as Antibiotic Carriers: Processing Parameters, Fabrication and Characterization. Pharmaceutics.

[131] Hinz B. (2007). Formation and function of the myofibroblast during tissue repair. J. Investig. Dermatol..

[132] Costa A.M.A., Peyrol S., Pôrto L.C., Comparin J.P., Foyatier J.L., Desmoulière A. (1999). Mechanical forces induce scar remodeling: Study in non-pressure-treated versus pressure-treated hypertrophic scars. Am. J. Pathol..

[133] Li-Tsang C.W.P., Feng B.B., Huang L., Liu X.S., Shu S., Chan Y.T.Y., Cheung K.K. (2015). A histological study on the effect of pressure therapy on the activities of myofibroblasts and keratinocytes in hypertrophic scar tissues after burn. Burns.

[134] He J.H., Fang B., Shan S.Z., Li Q.F. (2023). Mechanical stiffness promotes skin fibrosis through Piezo1-mediated arginine and proline metabolism. Cell Death Discov..

[135] Huang D., Shen K.H., Wang H.G. (2013). Pressure therapy upregulates matrix metalloproteinase expression and downregulates collagen expression in hypertrophic scar tissue. Chin. Med. J..

[136] Powell H.M., Nedelec B. (2022). Mechanomodulation of Burn Scarring Via Pressure Therapy. Adv. Wound Care.

[137] Zhao J.L., Yang S., Xu Y.B., Qin S.T., Bie F., Chen L., Zhou F., Xie J.L., Liu X.S., Shu B. (2023). Mechanical pressure-induced dedifferentiation of myofibroblasts inhibits scarring via SMYD3/ITGBL1 signaling. Dev. Cell.

[138] Wang T., Wu H., Liu S., Lei Z.J., Qin Z.Y., Wen L.Z., Liu K.J., Wang X.W., Guo Y., Liu Q. (2018). SMYD3 controls a Wnt-responsive epigenetic switch for ASCL2 activation and cancer stem cell maintenance. Cancer Lett..

[139] Chambers R.J., Rajput B.S., Scofield G.B., Reindel J., O’Shea K., Li R.J., Simkovsky R., Mayfield S.P., Burkart M.D., Cai S. (2024). Mechanically Robust and Biodegradable Electrospun Membranes Made from Bioderived Thermoplastic Polyurethane and Polylactic Acid. ACS Appl. Polym. Mater..

[140] Ricci C., Azimi B., Panariello L., Antognoli B., Cecchini B., Rovelli R., Rustembek M., Cinelli P., Milazzo M., Danti S. (2023). Assessment of Electrospun Poly(ε-caprolactone) and Poly(lactic acid) Fiber Scaffolds to Generate 3D In Vitro Models of Colorectal Adenocarcinoma: A Preliminary Study. Int. J. Mol. Sci..

[141] Alharbi N., Guthold M. (2024). Mechanical properties of hydrated electrospun polycaprolactone (PCL) nanofibers. J. Mech. Behav. Biomed. Mater..

[142] Gao X., Hou T., Wang L., Liu Y., Guo J., Zhang L., Yang T., Tang W., An M., Wen M. (2024). Aligned electrospun fibers of different diameters for improving cell migration capacity. Colloids Surf B Biointerfaces.

[143] Zhang Y., Wang X., Zhang Y., Liu Y., Wang D., Yu X., Wang H., Bai Z., Jiang Y.C., Li X. (2021). Endothelial Cell Migration Regulated by Surface Topography of Poly(ε-caprolactone) Nanofibers. ACS Biomater Sci Eng.

[144] Amer S.S., Mamdouh W., Nasr M., ElShaer A., Polycarpou E., Abdel-Aziz R.T.A., Sammour O.A. (2022). Quercetin loaded cosm-nutraceutical electrospun composite nanofibers for acne alleviation: Preparation, characterization and experimental clinical appraisal. Int. J. Pharm..

[145] Uhlírová R., Langová D., Bendová A., Gross M., Skoumalová P., Márová I. (2023). Antimicrobial Activity of Gelatin Nanofibers Enriched by Essential Oils against and. Nanomaterials.

[146] El-Naggar M.E., Abdelgawad A.M., Abdel-Sattar R., Gibriel A.A., Hemdan B.A. (2023). Potential antimicrobial and antibiofilm efficacy of essential oil nanoemulsion loaded polycaprolactone nanofibrous dermal patches. Eur. Polym. J..

[147] Tang Y., Liu L., Han J.J., Zhang Z.L., Yang S.Y., Li S.X., Fan Z.H., Zhao H. (2021). Fabrication and Characterization of Multiple Herbal Extracts-loaded Nanofibrous Patches for Topical Treatment of Acne Vulgaris. Fibers Polym..

[148] Khoshbakht S., Asghari-Sana F., Fathi-Azarbayjani A., Sharifi Y. (2020). Fabrication and characterization of tretinoin-loaded nanofiber for topical skin delivery. Biomater. Res..

[149] Rahnama S., Movaffagh J., Shahroodi A., Jirofti N., Bazzaz B.S.F., Beyraghdari M., Hashemi M., Kalalinia F. (2022). Development and characterization of the electrospun melittin-loaded chitosan nanofibers for treatment of acne vulgaris in animal model. J. Ind. Text..

[150] Karakucuk A., Tort S. (2020). Preparation, characterization and antimicrobial activity evaluation of electrospun PCL nanofiber composites of resveratrol nanocrystals. Pharm. Dev. Technol..

[151] Luo Y., Li B., Liu X.M., Zheng Y.F., Wang E.R., Li Z.Y., Cui Z.D., Liang Y.Q., Zhu S.L., Wu S.L. (2022). Simultaneously enhancing the photocatalytic and photothermal effect of NH-MIL-125-GO-Pt ternary heterojunction for rapid therapy of bacteria-infected wounds. Bioact. Mater..

[152] Jeong S., Oh S.G. (2022). Antiacne Effects of PVA/ZnO Composite Nanofibers Crosslinked by Citric Acid for Facial Sheet Masks. Int. J. Polym. Sci..

[153] Torres-Martínez E.J., Bravo J.M.C., Medina A.S., González G.L.P., Gómez L.J.V. (2018). A Summary of Electrospun Nanofibers as Drug Delivery System: Drugs Loaded and Biopolymers Used as Matrices. Curr. Drug Deliv..

[154] Chang P., Guo K., Li S., Wang H., Tang M. (2023). In Situ Sodium Chloride Cross-Linked Fish Skin Collagen Scaffolds for Functional Hemostasis Materials. Small.

[155] Kong X., Zhu D., Hu Y., Liu C., Zhang Y., Wu Y., Tan J., Luo Y., Chen J., Xu T. (2024). Melt electrowriting (MEW)-PCL composite Three-Dimensional exosome hydrogel scaffold for wound healing. Mater. Des..

[156] Ashrafi F., Emami A., Sefidbakht S., Aghayan H., Soleimani F., Omidfar K. (2025). Accelerated healing of full-thickness skin wounds by multifunctional exosome-loaded scaffolds of alginate hydrogel/PCL nanofibers with hemostatic efficacy. Int. J. Biol. Macromol..

[157] Kim J.I., Kim J.Y., Kook S.H., Lee J.C. (2022). A novel electrospinning method for self-assembled tree-like fibrous scaffolds: Microenvironment-associated regulation of MSC behavior and bone regeneration. J. Mater. Sci. Technol..

[158] Jin S., Luo Z., Cai Y., Wen J., Lu P., Fu X., Mou P., Chen A., Meng W., Li J. (2024). Exosome-functionalized heterogeneous nanofibrous scaffolds repair bone defects accompanied by muscle injury. Chem. Eng. J..

[159] Zhan J., Chen Z., Liu J., Pang Q., Lei M., Liu J., Song Y., Huang W., Dong L. (2025). A Targeting Trained Immunity Nanofiber Scaffold for Large Bone Defect Repair. Adv. Fiber Mater..

[160] Tang Y., Xu Z., Tang J., Xu Y., Li Z., Wang W., Wu L., Xi K., Gu Y., Chen L. (2023). Architecture-Engineered Electrospinning Cascade Regulates Spinal Microenvironment to Promote Nerve Regeneration. Adv. Healthc. Mater..

[161] Li J., Li X., Li X., Liang Z., Wang Z., Shahzad K.A., Xu M., Tan F. (2024). Local Delivery of Dual Stem Cell-Derived Exosomes Using an Electrospun Nanofibrous Platform for the Treatment of Traumatic Brain Injury. ACS Appl. Mater. Interfaces.

[162] Gopalarethinam J., Nair A.P., Iyer M., Vellingiri B., Subramaniam M.D. (2023). Advantages of mesenchymal stem cell over the other stem cells. Acta Histochem..

[163] Zakrzewski W., Dobrzynski M., Szymonowicz M., Rybak Z. (2019). Stem cells: Past, present, and future. Stem Cell Res. Ther..

[164] Zhou T., Yuan Z.N., Weng J.Y., Pei D.Q., Du X., He C., Lai P.L. (2021). Challenges and advances in clinical applications of mesenchymal stromal cells. J. Hematol. Oncol..

[165] Sundaramurthi D., Krishnan U.M., Sethuraman S. (2014). Electrospun Nanofibers as Scaffolds for Skin Tissue Engineering. Polym. Rev..

[166] Wu Y.J., Chen L., Scott P.G., Tredget E.E. (2007). Mesenchymal stem cells enhance wound healing through differentiation and angiogenesis. Stem Cells.

[167] Bose R.J.C., Kim B.J., Arai Y., Han I.B., Moon J.J., Paulmurugan R., Park H., Lee S.H. (2018). Bioengineered stem cell membrane functionalized nanocarriers for therapeutic targeting of severe hindlimb ischemia. Biomaterials.

[168] Liu H., Li R.C., Liu T., Yang L.Y., Yin G., Xie Q.B. (2020). Immunomodulatory Effects of Mesenchymal Stem Cells and Mesenchymal Stem Cell-Derived Extracellular Vesicles in Rheumatoid Arthritis. Front. Immunol..

[169] Lee D.E., Ayoub N., Agrawal D.K. (2016). Mesenchymal stem cells and cutaneous wound healing: Novel methods to increase cell delivery and therapeutic efficacy. Stem Cell Res. Ther..

[170] Wen Z.B., Chen Y.X., Liao P.L., Wang F.Y., Zeng W.P., Liu S.P., Wu H.B., Wang N., Moroni L., Zhang M.M. (2023). In Situ Precision Cell Electrospinning as an Efficient Stem Cell Delivery Approach for Cutaneous Wound Healing. Adv. Healthc. Mater..

[171] Liu N., Zhou Z.Y., Ning X.C., Zhang X.P., Guo Q.X., Guo M.X., Wang Y.F., Wu T. (2023). Enhancing the paracrine effects of adipose stem cells using nanofiber- based meshes prepared by light-welding for accelerating wound healing. Mater. Des..

[172] Sadtler K., Estrellas K., Allen B.W., Wolf M.T., Fan H.N., Tam A.J., Patel C.H., Luber B.S., Wang H., Wagner K.R. (2016). Developing a pro-regenerative biomaterial scaffold microenvironment requires T helper 2 cells. Science.

[173] Su N., Hao Y.Y., Wang F., Hou W.D., Chen H.F., Luo Y. (2021). Mesenchymal stromal exosome-functionalized scaffolds induce innate and adaptive immunomodulatory responses toward tissue repair. Sci. Adv..

[174] Xue J., Pisignano D., Xia Y. (2020). Maneuvering the Migration and Differentiation of Stem Cells with Electrospun Nanofibers. Adv. Sci..

[175] Kalluri R., LeBleu V. (2020). The biology function and biomedical applications of exosomes. Science.

[176] Deng D., Zhang J., Gan D., Zou J., Wu R., Tian Y., Yin Y., Li X., Chen F., He X. (2022). Roles of extracellular vesicles in periodontal homeostasis and their therapeutic potential. J. Nanobiotechnol..

[177] Lin J., Li J., Huang B., Liu J., Chen X., Chen X., Xu Y., Huang L., Wang X. (2015). Exosomes: Novel biomarkers for clinical diagnosis. Sci. World J..

[178] Mi B., Chen L., Xiong Y., Yang Y., Panayi A., Xue H., Hu Y., Yan C., Hu L., Xie X. (2022). Osteoblast/Osteoclast and Immune Cocktail Therapy of an Exosome/Drug Delivery Multifunctional Hydrogel Accelerates Fracture Repair. ACS Nano.

[179] Li Q., Hu W., Huang Q., Yang J., Li B., Ma K., Wei Q., Wang Y., Su J., Sun M. (2023). MiR146a-loaded engineered exosomes released from silk fibroin patch promote diabetic wound healing by targeting IRAK1. Signal Transduct. Target. Ther..

[180] Shiekh P.A., Mohammed S.A., Gupta S., Das A., Meghwani H., Maulik S.K., Banerjee S.K., Kumar A. (2022). Oxygen releasing and antioxidant breathing cardiac patch delivering exosomes promotes heart repair after myocardial infarction. Chem. Eng. J..

[181] Hu P., Armato U., Freddi G., Chiarini A., Dal Pra I. (2023). Human Keratinocytes and Fibroblasts Co-Cultured on Silk Fibroin Scaffolds Exosomally Overrelease Angiogenic and Growth Factors. Cells.

[182] Singh A., Shiekh P.A., Qayoom I., Srivastava E., Kumar A. (2021). Evaluation of polymeric aligned NGCs and exosomes in nerve injury models in diabetic peripheral neuropathy condition. Eur. Polym. J..

[183] Wang X.T., Chen J.L., Tian W.D. (2022). Strategies of cell and cell-free therapies for periodontal regeneration: The state of the art. Stem Cell Res. Ther..

[184] Zhu D.H., Johnson T.K., Wang Y., Thomas M., Huynh K., Yang Q.L., Bond V.C., Chen Y.E., Liu D. (2020). Macrophage M2 polarization induced by exosomes from adipose-derived stem cells contributes to the exosomal proangiogenic effect on mouse ischemic hindlimb. Stem Cell Res. Ther..

[185] Cheng P., Xie X., Hu L., Zhou W., Mi B., Xiong Y., Xue H., Zhang K., Zhang Y., Hu Y. (2024). Hypoxia endothelial cells-derived exosomes facilitate diabetic wound healing through improving endothelial cell function and promoting M2 macrophages polarization. Bioact. Mater..

[186] Wang Y.L., Lin Q.S., Zhang H., Wang S.C., Cui J., Hu Y., Liu J.L., Li M.M., Zhang K., Zhou F.J. (2023). M2 macrophage-derived exosomes promote diabetic fracture healing by acting as an immunomodulator. Bioact. Mater..

[187] Guan P.F., Liu C., Xie D.H., Mao S.C., Ji Y.L., Lin Y.C., Chen Z., Wang Q.Y., Fan L., Sun Y.J. (2022). Exosome-loaded extracellular matrix-mimic hydrogel with anti-inflammatory property Facilitates/promotes growth plate injury repair. Bioact. Mater..

[188] Zhao S.X., Kong H.R., Qi D.H., Qiao Y.S., Li Y., Cao Z.M., Wang H.W., He X.F., Liu H.D., Yang H. (2025). Epidermal stem cell derived exosomes-induced dedifferentiation of myofibroblasts inhibits scarring via the miR-203a-3p/PIK3CA axis. J. Nanobiotechnol..

[189] Li Y., Zhang J., Shi J., Liu K., Wang X., Jia Y., He T., Shen K., Wang Y., Liu J. (2021). Exosomes derived from human adipose mesenchymal stem cells attenuate hypertrophic scar fibrosis by miR-192-5p/IL-17RA/Smad axis. Stem Cell Res. Ther..

[190] Yao X.D., Cui X.M., Wu X.Y., Xu P., Zhu W.Y., Chen X.D., Zhao T.L. (2018). Tumor suppressive role of miR-1224-5p in keloid proliferation, apoptosis and invasion via the TGF-β1/Smad3 signaling pathway. Biochem. Biophys. Res. Commun..

[191] Jin M.Z., Xu X. (2023). MicroRNA-182-5p Inhibits Hypertrophic Scar Formation by Inhibiting the Proliferation and Migration of Fibroblasts via SMAD4 Pathway. Clin. Cosmet. Investig. Dermatol..

[192] Zhao W., Zhang R., Zang C.Y., Zhang L.F., Zhao R., Li Q.C., Yang Z.J., Feng Z., Zhang W., Cui R.T. (2022). Exosome Derived from Mesenchymal Stem Cells Alleviates Pathological Scars by Inhibiting the Proliferation, Migration and Protein Expression of Fibroblasts via Delivering miR-138-5p to Target SIRT1. Int. J. Nanomed..

[193] Wu D., Liu X., Jin Z. (2023). Adipose-derived mesenchymal stem cells-sourced exosomal microRNA-7846-3p suppresses proliferation and pro-angiogenic role of keloid fibroblasts by suppressing neuropilin 2. J. Cosmet. Dermatol..

[194] Yam G.H.-F., Santra M., Sahel J.A., Funderburgh J.L., Jhanji V. (2022). Human corneal stromal stem cells express microRNA-29a in exosomes—A robust cell selection for stem cell therapy of corneal scarring. Investig. Ophthalmol. Vis. Sci..

[195] Jian X., Qu L., Wang Y., Zou Q., Zhao Q., Chen S., Gao X., Chen H., He C. (2019). Trichostatin A-induced miR-30a-5p regulates apoptosis and proliferation of keloid fibroblasts via targeting BCL2. Mol. Med. Rep..

[196] Liu P., Hu Y., Xia L., Du M., Hu Z. (2020). miR-4417 suppresses keloid fibrosis growth by inhibiting CyclinD1. J. Biosci..

[197] Wang R., Bai Z., Wen X., Du H., Zhou L., Tang Z., Yang Z., Ma W. (2019). MiR-152-3p regulates cell proliferation, invasion and extracellular matrix expression through by targeting FOXF1 in keloid fibroblasts. Life Sci..

[198] Pang Q., Wang Y., Xu M., Xu J., Xu S., Shen Y., Xu J., Lei R. (2019). MicroRNA-152-5p inhibits proliferation and migration and promotes apoptosis by regulating expression of Smad3 in human keloid fibroblasts. BMB Rep..

